# Pharmacoinformatics, Adaptive Evolution, and Elucidation of Six Novel Compounds for Schizophrenia Treatment by Targeting DAOA (G72) Isoforms

**DOI:** 10.1155/2017/5925714

**Published:** 2017-01-19

**Authors:** Sheikh Arslan Sehgal

**Affiliations:** ^1^Department of Biosciences, COMSATS Institute of Information Technology, Sahiwal, Pakistan; ^2^State Key Laboratory of Biomembrane and Membrane Biotechnology, Institute of Zoology, Chinese Academy of Sciences, Beijing, China; ^3^University of Chinese Academy of Sciences, Beijing, China

## Abstract

Studies on Schizophrenia so far reveal a complex picture of neurological malfunctioning reported to be strongly associated with DAOA. Detailed sequence analyses proved DAOA as a primate specific gene having conserved gene desert region on both upstream and downstream region. The analyses of 10 MB chromosomal region of primates, birds, rodents, and reptiles having DAOA evidenced the conserved part in primates and in the rest of species, while* DAOA* is only present in primates. DAOA has four isoforms having one interaction partner DAO. Protein-protein analyses of four DAOA isoforms with DAO were performed individually and find potential interacting residues computationally. It was observed that molecular docking of approved FDA drugs revealed efficient results but there was no common drug with effective binding to all DAOA isoforms. Library of compounds was constructed by virtual screening of 2D similarity search against recommended SZ drugs in conjunction with their physiochemical properties. Molecular docking resulted in six novel compounds exhibiting maximum binding affinity with selected four DAOA isoforms. However not the entire schizophrenic population responds to the single drug and interestingly in this study six novel compounds having promising results and same binding site to that DAOA that may be used to interact with DAO against four DAOA isoforms were observed.

## 1. Introduction

Schizophrenia (SZ) affects about 1% of the population of world showing similar prevalence throughout sundry ethnic groups [1]. It is a highly heritable, chronic mental, and widespread disease characterized by neuropsychological abnormalities and neurophysiology impairment [1–3]. SZ vulnerability is influenced by polygenic components, environment factors, and their interactions [4]. The molecular mechanisms that activate SZ are still unclear. The identification of SZ genes is particularly demanding and exigent due to limited SZ diagnosis accuracy as phenotypic definition and various entities that have not been yet defined. Furthermore, the lack of conclusive genome scan linkage could be due to the existence of numerous SZ susceptibility genes that are difficult to detect and replicate [5].

The variations in D-amino acid oxidase activator* (DAOA)* (13q34) gene initially were linked with SZ [6]. Additionally,* DAOA* has been associated with other phenotypes and psychiatric disorders like major depression [7] and bipolar disorder [8]. The genetic variations of* DAOA* were contributed to numerous CNS disorders associated with glutamatergic signaling dysfunction [6, 9, 10]. The canonical ORF of G72 (DAOA) is predicted to encode a putative protein of 153 amino acids isolated from amygdale libraries, caudate nucleus, spinal cord, and testis [6]. The expression of* DAOA* in transgenic mice induced schizophrenic related behavioral phenotypes [11, 12].

The overexpression of DAOA in schizophrenic patients has been reported in dorsolateral prefrontal cortex in parallel to healthy controls [13]. The vulnerability of SZ genes has been identified in various genetic studies [14–17], but genetic interactions and their interplay among SZ genes with neurobiological abnormalities and clinical subtypes are still unclear. An enzyme is the product of* DAO* that degrades the D-serine amino acid which acts as coagonist at the glycine site of the N-methyl-D-aspartic acid (NMDA) receptors [18]. The* DAOA* product activates the DAO enzyme [6]. The biological functions of* DAO* and* DAOA* are engrossed in the hypothesized hypofunction of NMDA receptor complex as the prospective pathogenesis of SZ [19].

The NMDA neurotransmission has dominant molecular mechanism for synaptic plasticity, cognition, and memory. Several neurological and psychiatric disorders are associated with dysfunction of NMDA receptor mediated neurotransmission [20]. Overexpression and hyperactivity of brain DAO have been linked with SZ [21, 22].

There has been much progress in personalized medicine and computational drug designing from last decade and more opportunities are available to understand neurological diseases. Various biological problems have been solved by employing bioinformatics approaches [23] and structural bioinformatics have effective methodologies to design active novel compounds against neurological disorders [24–27] and cancer [28, 29]. It has been reported that diethoxyphosphinothioyl (2E)-2-(2-amino-1, 3-thiazol-4-yl)-2-trityloxyiminoacetate (C_28_H_28_N_3_O_5_PS_2_) is efficacious in the SZ treatment for targeting DAOA [25]. In silico analyses of DAOA isoforms have higher probability and efficacy on the basis of binding energy. C_28_H_28_N_3_O_5_PS_2_ was reported as potent inhibitor against DAOA-125 (accession number A2T115) for inhibition of SZ [23]. Another study reported C_28_H_28_N_3_O_5_PS_2_ as significant inhibitor against 4 DAOA isoforms [25]. The efforts were initiated with the extensive literature review regarding DAOA and SZ disorder. The objective of this work was (1) computational sequence analyses of primates, birds, rodents, and reptiles, (2) comparative phylogenetic analyses and 10 MB chromosomal region comparative analyses of primates, birds, rodents, and reptiles, (3) 3D structure prediction of selected DAOA isoforms and evaluations, (4) comparative pharmacoinformatics analyses of recommended drugs for SZ, (5) generation of ligand-based pharmacophore and virtual screening, (6) identification of novel hits against SZ by targeting DAOA isoforms, and (7) protein-protein interaction studies. To accomplish these objectives, sequence analyses, comparative evolutionary analyses, homology modeling and threading based approaches, pharmacoinformatics analyses, comparative molecular docking approaches, and ADMET drug properties were utilized followed by numerous structural bioinformatics and comparative genomics analyses. The results confirmed that followed strategies were capable of identifying the effective drug analog among the recommended drugs and also the identification of novel inhibitors for SZ by targeting DAOA isoforms.

## 2. Materials and Methods

The* DAOA *transcribed 2 different transcripts that form four isoforms collectively as DAOA-82, DAOA-125, DAOA-126, and DAOA-153. The DAOA-125 has the accession number A2T115 and rest of the three isoforms (DAOA-82, DAOA-126, and DAOA-153) have single accession number P59103 in Uniprot Knowledge Base. In present work, sequence analyses, synteny analyses, 3D structure prediction, comparative molecular docking studies, and comparative pharmacoinformatics analyses were performed.

### 2.1. Sequence Analyses

The ENSEMBL (http://asia.ensembl.org/index.html) and UCSC (https://genome.ucsc.edu/) Genome browsers were utilized for sequence analyses of primates, rodents, birds, and reptiles and for generating the synteny of* DAOA*. MEGA5 [30] tool was used for constructing the phylogenetic trees and bootstrap values were also calculated and analyzed.

### 2.2. Structure Prediction

The amino acid sequences of DAOA isoforms were retrieved from Uniprot KB (http://www.uniprot.org/) and were subjected to BLASTp for the identifications of suitable templates against Protein Data Bank (PDB) [31]. The protein modeling automated program MODELLER 9.14 [32] for comparative homology modeling was employed to predict three-dimensional (3D) structures of DAOA by satisfying spatial restraints. Threading approach (SWISS MODEL [33], I-TASSER [34], MOD-WEB [35], 3D-JigSaw [36], and ESyPred3D [37]) were also employed for structure prediction. The 3D structures for DAOA isoforms were visualized on UCSF Chimera 1.10. The predicted structures of DAOA isoforms were minimized by AMBER [38] software. The structures were evaluated by MolProbity [39]. The poor ramachandran outliers and rotamers were removed by utilizing WinCoot [40] tool. Rampage [41], ProCheck [42], Anolea [43], and ERRAT [44] were used for the overall assessment of protein structure verifications and model quality. The generated ramachandran plots for evaluation of predicted models showed residues distribution and also revealed Φ and Ψ distributions of non-Glycine, non-Proline residues. The psi and phi angles were plotted against each other to differentiate the unfavorable and favorable regions. These angles were utilized to evaluate the quality of regions. Two lines were drawn on the error axis for the confidence to reject the regions that exceed the error value and the percentage of the protein for calculating the error value falls below the 95% of rejection limit. Generally, high resolution structures produce values above form 95%. Errat evaluations tool was utilized to calculate the overall quality factors of all the predicted structures. The energy minimization was also done for further structure refinement through UCSF Chimera 1.10 [45].

### 2.3. Pharmacophore Generation

The pharmacophore was generated by using LigandScout 3.1 [46] and drugs were employed in the ligand-based module of LigandScout. Pharmacophoric sites (hydrogen bond donor, hydrogen bond acceptor, hydrophobic sites, aromatic rings, and positive and negative groups) were analyzed. To incorporate all the selected features of drugs, merge feature model generation and atom overlap scoring function were used from ligand-based module of LigandScout 3.1. By utilizing the correct parameters, virtual screening (VS) shortens the inhibitor search time by screening large databases (Drug-Like, 20000 Compounds, and Drug). The VS was performed by using LigandScout alignment and screening modules.

### 2.4. Comparative Docking

The binding residues were investigated by employing Site Hound, Q-site finder, and Computed Atlas of Surface Topography of Proteins (CASTp) [47, 48]. The geometry optimization and energy minimization of known and novel molecules were performed by Chem3D Ultra [49] and UCSF Chimera 1.10. The comparative molecular docking studies were carried out by utilizing Genetic Optimization for Ligand Docking (GOLD) [50], AutoDock Vina [51], and AutoDock 4.0 [52]. The automated docking was performed by employing the AutoDock 4.0 tools to locate the suitable binding conformations and binding orientations of drugs and ligands. The selected drugs and scrutinized ligands were docked by selected docking tools and results were further analyzed in conjunction with the results by AutoDock tools by employing UCSF Chimera 1.10. Ligplot 2 [53] and UCSF Chimera 1.10 were used to visualize, analyze, and identify the interactions.

### 2.5. ADMET Properties

The number of H-bond donors, H-bond acceptors, and rotatable bonds were analyzed by utilizing molinspiration (http://www.molinspiration.com/) and mCule [54]. ADMET properties were evaluated by utilizing admetSAR online server [55]. The online tool Osiris Property Explorer [56] was utilized to estimate their possible reproductive and tumorigenic risks and also to calculate the drug score and drug-like properties of selected drugs and novel compounds. Rule of five was calculated by mCule server. The Osiris programs and mCule were used to estimate the mutagenesis of molecules.

### 2.6. Protein-Protein Docking Studies

STITCH4 (Search Tool for InTeracting CHemicals) [57] and STRING 10 (Search Tool for the Retrieval of INteracting Genes/Proteins) [58] were employed to analyze the functional partners of DAOA isoforms. The crystal structure of DAO (PDB ID: 2DU8) was retrieved from PDB. Gramm-X online server [59] was applied for protein docking studies of DAOA isoforms with interacting partner DAO. PatchDock [60] was employed to crosscheck and validation of the generated protein-protein interaction results. Afterwards, hydrophobic and electrostatic interactions were mapped by using LigPlot.

## 3. Results and Discussion

The field of structural bioinformatics, precision medicine, and neurosciences are blooming and the potential in SZ treatments is vivid. Besides, the research resources are devoted for the understanding of SZ and numerous scientists are trying to explore the effective treatment of SZ. DAOA, the SZ-related protein, plays significant role in the regulation of DAO in great apes and SZ associated with overexpression of DAOA and DAO. The hyperfunction of DAOA leads to the upregulation of DAO activity which decreases the level of serine [25]. The abnormal level of D-serine may result in SZ and elaborative pharmacoinformatics, protein-ligand, and protein-protein interaction studies demonstrated that the C-terminal of DAOA can regulate DAO and NMDA neurotransmission. It is a dominant molecular mechanism for memory, cognition, and synaptic plasticity. Various psychiatric and neurological diseases are linked with dysfunction of NMDA [20]. The hyperfunction of DAOA in brain has been linked with SZ and leads to the hyperactivity of DAO resulting in decreasing the level of D-serine and hypofunction of NMDA [25]. The significance and contribution of DAOA in various CNS diseases linked with glutamatergic signaling dysfunction [6, 9, 10] and the expression of DAOA could provide potential therapeutic benefits. The inhibitors of DAOA may give a valuable therapeutics strategy to treat SZ. The four isoforms of DAOA were analyzed and a functional conserved C-terminal region was revealed in all the utilized four DAOA isoforms and proposed that the revealed region showed significance for DAOA folding and function. The results considered as the landmark and provide better significant understanding of DAOA. The analyses determined the interacting domain of DAOA and, by utilizing in silico approaches, demonstrated that DAOA interact via C-terminal. The common interacting residues of C-terminal from all the selected four isoforms of DAOA which interact with drugs, novel inhibitors, and DAO may have significance to treat SZ.

### 3.1. Sequence and Phylogenetic Analyses

Examination of extensive literature and biological database entries revealed numerous interesting sequence information of DAOA. Protein sequence data sets for enormous range of invertebrates and vertebrates genomes are available for analyzing the protein sequence analyses of DAOA. EnsEMBL BLAT and NCBI BLAST tools were utilized against NCBI for sequence alignment of DAOA canonical protein sequence against all present ENSEMBL species. It was argued previously by Sehgal et al. (2015) that DAOA is a primate specific gene and they analyzed the available biological data (March 2014) [25]. The identity and query coverage of analyzed species were analyzed (Table 1) and BLOSSUM62 matrix was utilized for scoring having a 3-word size and expected threshold of 10. To investigate more critically, the word size of 2 was also employed and no variation was observed in results according to the analyses of currently available (July 27, 2016) biological sequences data in biological databases (NCBI, ENSEMBL, and UCSC). The in silico sequence analyses revealed that* DAOA* was only present in humans, chimpanzees, gorillas, orangutans, and crab-eating macaques.

**Figure 1:**
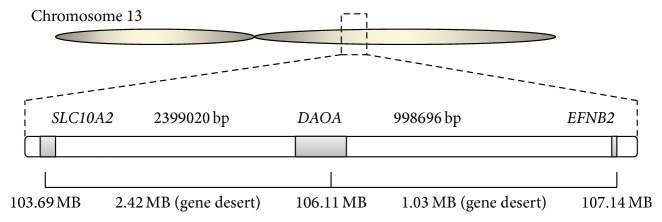
Gene desert on both regions of* DAOA* in human chromosome 13.

**Figure 2:**
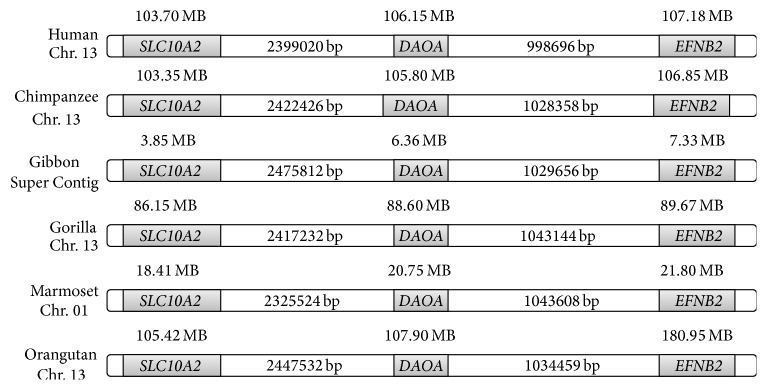
Conserved gene desert in analyzed species.

The* DAOA* located on chromosome 13 in humans and gene desert was observed in upstream and downstream regions of* DAOA* (Figure 1). The interesting fact was observed that the gene desert was conserved in species which have* DAOA* (Figure 2). The* EFNB2* gene was observed at the upstream region and* SLC10A2* gene was observed on the downstream region. The genomes of mouse (rodent), chicken (birds), and lizard (reptiles) were also analyzed critically regarding* DAOA* and observed the absence of* DAOA* in rodents, birds, and reptiles. The interesting observation was the presence of gene desert on chromosomal location of* DAOA* in mouse, chicken, and lizard. The upstream gene* (EFNB2)* and downstream gene* (SLC10A2)* were observed in rodents, birds, and reptiles as were investigated in human (Figure 2).

**Figure 3:**
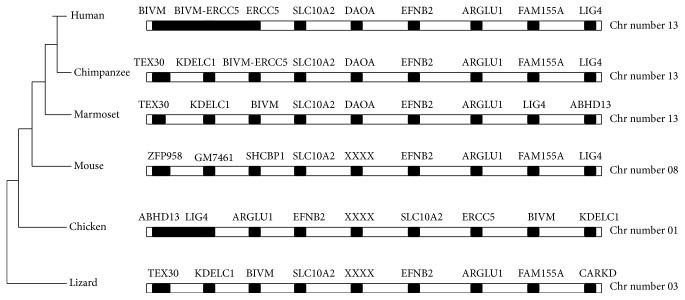
Phylogenetic tree of DAOA constructed by neighbor-joining (NJ) method and absence of DAOA in rodents, chicken, and reptiles.

The phylogenetic tree constructed by neighbor-joining (NJ) method (Figure 3) revealed the lineage of* DAOA* and absence in rodents, birds, and reptiles. It was observed that* DAOA* is inserted in great apes about 35 million years ago before the divergence of new world monkeys from old world monkeys. The synteny of human, chimpanzee, gibbon, gorilla, marmoset, and orangutan were also analyzed by utilizing ENSEMBL genome browser. The 5 Mb chromosomal regions from both downstream and upstream of* DAOA* were analyzed in species having* DAOA* and gene desert was conserved on both regions. The* DAOA* was observed as the conserved region in primates and absent in all other species. The insertion of* DAOA* in primate's genome is still unclear. The 10 MB chromosomal regions of analyzed species were observed and conserved and also conservation in birds, rodents, and reptiles was also found (see Supplementary Figure  1 in Supplementary Material available online at https://doi.org/10.1155/2017/5925714).

### 3.2. Structure Prediction

The 3D structures of DAOA isoforms were not reported yet by X-ray crystallography and NMR techniques. Comparative homology modeling and threading approaches were utilized to predict the 3D structures of DAOA isoforms. The sequences of DAOA isoforms were subjected to BlastP against PDB database for the search of suitable templates. The top ranked five optimally aligned suitable templates with query coverage, maximum identity,* E* values, and total scores were selected for comparative homology modeling. Sequence alignment of protein residues showed that the conserved part in sequence will have the similar functions. The scrutinized templates were utilized to generate 3D structures of DAOA isoforms. The overall query coverage and similarity among the utilized templates and DAOA isoforms showed >45% from end to end that was not considered satisfactory for reliable structures by homology modeling approach. To overcome the errors and for better 3D structure, threading approach was utilized.

Numerous models of DAOA isoforms were predicted by utilizing various tools (I-TASSER, M4t, Mod Web, SWISS MODEL, HHpred, Phyre2, intFOLD2, 3D-jigsaw, and MODELLER 9.14) and in silico approaches (homology modeling and threading) by satisfying the sequence.

**Figure 4:**
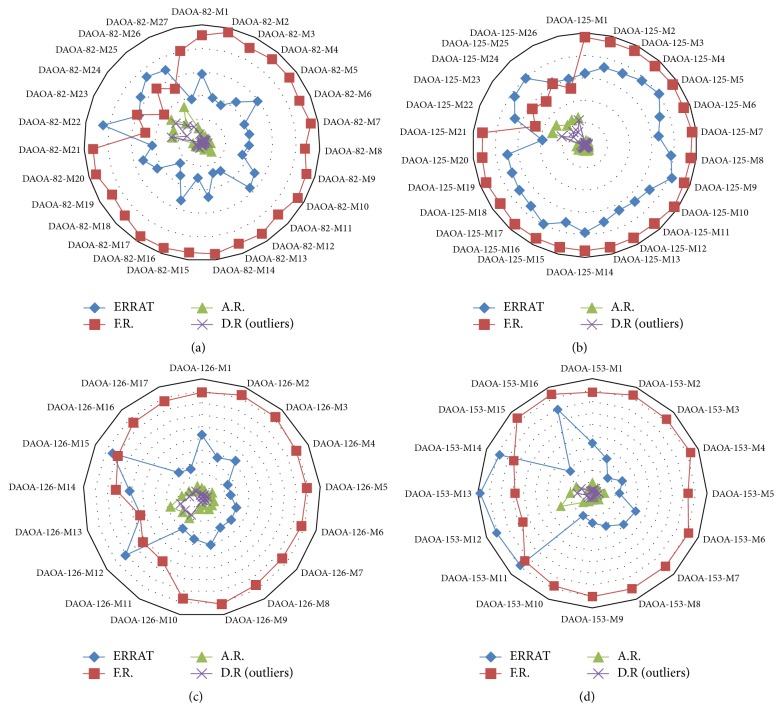
Comparative evaluation analyses of generated 3D models based on ERRAT quality factor (blue line), favored region (red line), allowed region (green line), and outliers (purple line).

All the generated models were evaluated on the basis of favored region, allowed region, outliers, overall quality factor (Supplementary file 1), and binding regions. The generated comparative graphs (Figure 4) of all the predicted models favor the model generated from threading approach. The most reliable structures were selected from the generated graphs. The predicted 3D structures of DAOA isoforms were simulated for 20 nanoseconds by utilizing the AMBER software.

**Figure 5:**
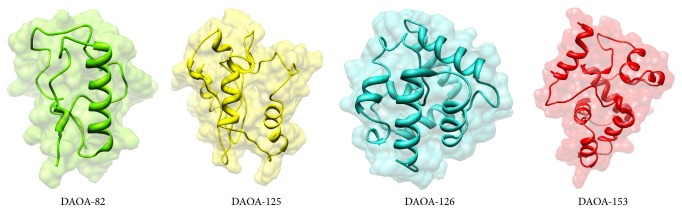
3D structure of DAOA isoforms.

ERRAT showed overall quality factor of 91.892% in DAOA-82, 96.581% in DAOA-125, 94.915% in DAOA-126, and 91.7324% in DAOA-153 (Supplementary File 1), depicting the high quality of structures. The energy minimization on optimal predicted structures of DAOA isoforms was applied to improve the stereochemistry furthermore and the most optimal models were considered for this purpose. The selected structures after the critical examining of evaluation parameters were subjected to UCSF Chimera 1.10 for minimization at 1000 steepest and conjugates gradients runs. The selected minimized structures of DAOA isoforms (Figure 5) have the potential of employing for further drug analyses against known and novel compounds.

### 3.3. Comparative Molecular Docking Studies

**Figure 6:**
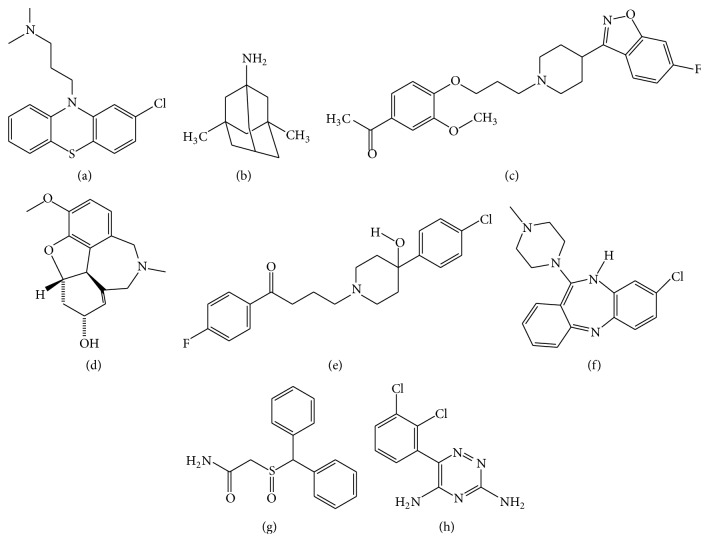
Two-dimensional structures of selected drugs. (a) Chlorpromazine, (b) Memantine, (c) Iloperidone, (d) Galantamine, (e) Haloperidol, (f) Clozapine, (g) Modafinil, and (h) Lamictal.

The experimental analyses elucidated that the selected drug molecules (Figure 6) in present study have significant values for the treatment of SZ. However, the docking analyses of scrutinized drugs revealed variations in their binding energies and performed with 200 runs and all the generated docking complexes were saved, out of which the best complex showed interaction in binding pocket, having repeated binding residues and least binding energy was selected for each drug compound. The results indicated that the selected eight drug compounds (Chlorpromazine, Clozapine, Galantamine, Haloperidol, Iloperidone, Lamictal, Memantine, and Modafinil) effectively bind to DAOA isoforms (Table 2) and showed effective binding residues (Table 3).

The scrutinized eight drugs were also analyzed on the basis of drug properties, carcinogenicity, binding energy, and toxicity (Table 4). The compounds have cyclic molecules having significant biological properties. Docking analyses were done against all the selected eight drugs by utilizing GOLD docking software and crossvalidate the results by utilizing AutoDock and AutoDock Vina docking tools. All the utilized drugs showed effective results and it was observed that not a single drug was able to show effective results against all DAOA isoforms. The least binding energy and comparative analyses of utilized docking tools (AutoDock4, AutoDock Vina, and GOLD) observed that Galantamine for DAOA-82, Clozapine for DAOA-125, Iloperidone for DAOA-126, and Haloperidol for DAOA-153 were effective specifically. Not a single drug effectively bound with the selected four isoforms of DAOA while this observation leads to personalized medicine for better health and effective cure.

All the 8 selected drugs and the reported ligand molecule (C_28_H_28_N_3_O_5_PS_2_) [23, 25] were utilized to generate the pharmacophore models. Pharmacophoric sites including positive and negative ionizable groups, aromatic ring, hydrophobic sites, hydrogen bond acceptor (HBA), and hydrogen bond donor (HBD) were characterized carefully. Atoms overlap scoring function and merge feature model generation parameters were utilized to incorporate the associated features of drugs. Subsequently, the libraries (20,000 compounds, Drug, and Drug-Like) were screened by using LigandScout. After screening all the selected libraries, total 114 molecules were observed in the result of virtual screening that satisfies the characteristics of generated ligand-based pharmacophore.

**Figure 7:**
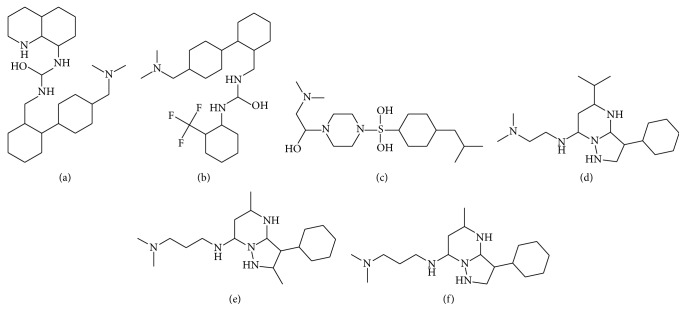
2D structure of scrutinized 6 novel molecules: (a) SA-1, (b) SA-3, (c) SA-11, (d) SA-68, (e) SA-110, and (f) SA-111.

The comparative docking studies were performed on the screened 114 molecules by utilizing the selected docking tools. All the generated complexes were ranked on the bases of least binding energy, highest binding affinity, and drug properties. The top 20 docked molecules from each utilized tools (AutoDock4, AutoDock Vina, and GOLD) were critically analyzed. Surprisingly, it was observed that novel molecules (SA-1, SA-3, SA-11, SA-68, SA-110, and SA-111) (Figure 7) from scrutinized 114 compounds were included in top 20 compounds of each tool and showed least bonding energies (Table 5) and effective binding affinity through AutoDock4, AutoDock Vina, and GOLD. The interesting fact was observed that the scrutinized top ranked molecules showed effective least binding energy against DAOA isoforms which the FDA approved drug analogs could not.

The entire screened novel compounds (114) and utilized drug analogs (08) bound on almost same binding region of their appropriate DAOA isoforms. In an effort to explore, the top six molecules scrutinized from all the 114 compounds screened from all the selected libraries were elucidated. The binding site analyses of DAOA isoforms were also revealed by employing SiteHound and CASTp. It was observed that the binding domains predicted by SiteHound were similar to the pocket revealed in molecular docking analyses and the measurements of binding pockets were also analyzed (Supplementary file 2).

The novel molecules may be considered as potential antischizophrenic agents. GOLD, AutoDock Vina, and AutoDock tools were employed to collective common complexes of drugs analyses and novel molecules of DAOA isoforms having effective drug properties (Table 6) and least binding energy were analyzed. The slight fluctuation was observed in analyzed complexes of DAOA isoforms having lowest binding energies. It was observed that scrutinized molecules bound at the conserved C-terminal region of DAOA isoforms and revealed the binding domain.

**Figure 8:**
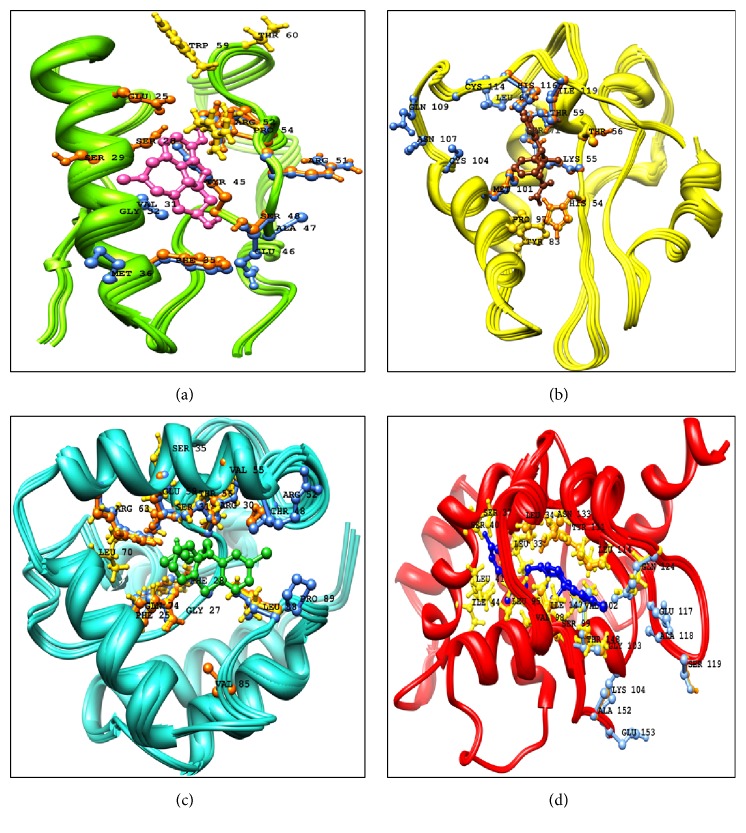
The DAOA isoforms interactions with appropriate drugs. The residues analyzed from AutoDock 4 were represented with orange, gold residues with gold, and AutoDock Vina with cornflower blue color. (a) Galantamine interaction with DAOA-82. (b) Modafinil interaction with DAOA-125. (c) Chlorpromazine interacting residues with DAOA-126. (d) Haloperidol with DAOA-153.

It was also observed that Ser-99 of DAOA-153, Ser-28 of DAOA-82, and Ser-71 of DAOA-125 showed good binding interactions and have different positions due to variation in the size of isoforms. The conserved region in DAOA isoforms behaved as binding domain but has different positions due to different size of isoforms. To visualize better interactions between amino acid and ligand residues in the active site of protein, a plot of ligand-protein interactions were generated by utilizing UCSF Chimera 1.10 (Figure 8).

### 3.4. ADMET and Drug Properties

The chemical structures of compounds are evaluated for oral bioavailability and to be an effectual drug compound subjected to Lipinski's rule of five [61]. The admetSAR online server was employed for absorption, distribution, metabolism, excretion, and toxicity (ADMET) properties of compounds. Mathematical models including Rat Acute Toxicity, human intestinal absorption, cytochrome P450 2D6 inhibition, acute oral toxicity, Caco2 Permeability, Honey Bee Toxicity, aqueous solubility (LogS), Fish Toxicity, blood–brain barrier penetration, and AMES Toxicity parameters were utilized to predict the ADMET properties of compounds. The prediction of different toxicities were often utilized in drug designing. These analyzed toxicities help in evaluating pollutants, metabolites, and intermediates along with adjusting the range of dose for animal assay.

The prediction of aqueous solubility (defined water at 25°C) of scrutinized molecules indicated that the selected compounds are soluble in water. The ratio of compound in octanol compared to its solubility in water is known as Lipophilicity (LogP) measurement solubility. It was concluded that molecules follow Lipinski's rule of five and revealed less LogP values involved in better oral bioavailability. The excretion process by which the body eliminates the drug molecule from body depends on LogP [61]. The drug molecules must be absorbed by human intestine and our generated results depict that the reported compounds can easily be absorbed by human intestine. The analyzed molecules were found to be noninhibitor of cytochrome P450 2D6, which indicated that analyzed molecules may be well metabolized in Phase I metabolism. The cytochrome P450 2D6 was always considered as key enzyme involved in the metabolism of drugs.

Toxicity risk assessment and carcinogenicity were analyzed for the scrutinized molecules and the analyses showed that all the analyzed molecules behave as noncarcinogenic. The analyses revealed that the reported residues are decisive and the mutational analyses of these binding residues could be effective. It also stands that the reported top 6 novel molecules in analyses have the tendency to be effective candidate for SZ treatment by targeting* DAOA*.

### 3.5. Protein-Protein Interactions

The DAOA was expressed in amygdala, caudate nucleus, spinal cord, and testis and revealed the binding domain at C-terminal in current analysis. DAO, the interacting partner of DAOA [23], was utilized for protein-protein docking studies. The protein-protein (DAOA-DAO) and the ligand-protein (selected compounds with DAOA) comparative molecular docking analyses were performed separately to check the residual involvement. The docked complex of DAOA-DAO predicted the interacting residues and their importance in the hyperfunction of DAO. The protein-protein docking analyses were performed and analyzed on the basis of approximate interface area of complex and Atomic Contact Energy (ACE) by utilizing PatchDock (Table 7). The 200 DAOA-DAO complexes were analyzed on the basis of ACE and top 10 complexes (Supplementary File 3) having least ACE values were scrutinized for further refinement and analyses by employing FireDock. The complexes were analyzed on the basis of least binding global energy, Attractive and Repulsive VdW (the contribution of the Van der Waals forces to the global binding energy), ACE, and HB (the contribution of the hydrogen bonds to the global binding energy). The DAOA82-DAO, DAOA125-DAO, DAOA-126, and DAOA153-DAO complexes showed the least global binding energy −15.65, −14.97, −22.87, and −29.01, respectively (Table 7). The least binding values suggested that DAOA and DAO have effective binding affinity due to which the complex may have capacity to regulate the downexpression to normalize the level of D-serine.

The genome and its related information study can shed light on numerous questions linked with the disease and health of an organism. Due to the completion of Human Genome Project (HGP), the DNA whole-genome sequence information is accessible for study and analyses. Personalized or precision medicine must be designed for whom the successful disease management rate is very low and for those patients who are not responding to traditional medicines. Personalized medicines are specified to the patients after analyzing genomic and proteomics information including the study of RNA and numerous metabolites considered as crucial factors for personalized medicine in medical decision making.

Every individual's genome or protein responds differently to injected molecule, drug, and medicine. The gene expression modifications are proscribed by epigenetic fundamental mechanisms as microRNA's, chromatin remodeling, histone modifications, DNA methylations, and RNA splicing. RNA splicing generates various variants of same protein and variants of single protein present in different populations and individuals. Every variant has different amino acids length and have different response for drugs and medicine. These influenced environmental changes may result in severe diseases and patients having different variants and alterations do not respond to traditional conventional medicines and therapies. Hence, the drugs for personalized medicine can be utilized to cure these diseases based on personal proteomics and genomics profiles of individuals. FDA also approved various drugs utilized for personal medicine. Every patient is unique due to its unique genome and proteome and exon shuffling also lead to showing difference. The four different variants of DAOA are utilized to reveal the binding pocket and lead to personalized medicine. The variants are present in different individuals and respond differently and scrutinized molecules may cure the SZ by targeting DAOA isoforms. This in silico approach will reduce the time phase and helped the researchers working on personalized medicine. It has been suggested that the manipulation of DAOA can be utilized for the treatment of SZ. The docking analyses provide elementary cues for synthesizing the reported molecules in this study and also for designing more potent molecules to cure SZ. The importance of DAO regulation in the neurology function has been revealed while the exact authenticated mechanism is still unclear. DAOA molecular characterization is reported as endogenous modulator of DAO activity. The study elucidates the binding interaction of DAOA isoforms with FDA approved drugs and novel molecules. By utilizing in silico and computational approaches, the conserved C-terminal region in DAOA isoforms has been revealed. The analyzed drugs and novel molecules showed binding residues in conserved C-terminal region of DAOA isoforms by GOLD, AutoDock4, and AutoDock Vina. This study also identified the common binding residue site and hypothesized that these residues have crucial role to normalize the expression of DAOA. The in silico analyses proposed that binding residues within C-terminal of DAOA are significant to control the expression instead of N-terminal. The results proposed that reported molecules could be used for novel chemical compounds. The synthetic peptides could also reduce the overexpression of DAOA.

## Supplementary Material

Analyses of 10 MB region of selected species and synteny of DAOA.

## Figures and Tables

**Table 1:** Identity and query coverage of primates with human.

Species name	Amino acid length (a.a)	Identity	Query coverage
Chimpanzee	146	98%	93%
Gibbon	112	73%	54%
Gorilla	149	96%	96%
Marmoset	61	74%	29%
Orangutan	148	92%	89%

**Table 2:** Comparative docking analyses of selected drugs.

Proteins	Drugs	Gold score	Estimated free energy of binding (kcal/mol)		Ligand efficiency	Estimated inhibition constant, Ki (*µ*M)	Final intermolecular energy (kcal/mol)
			AutoDock4	AutoDock-Vina			
DAOA-82	Chlorpromazine	—	−5.31	−6.6	−0.25	127.64	−6.07
	Clozapine	30.72	−5.31	−7.3	−0.23	128.12	−5.35
	*Galantamine*	*39.67*	−*5.36*	−*7.0*	−*0.27*	*118.72*	−*5.94*
	Haloperidol	40.09	−4.78	−7.2	−0.18	316.13	−6.79
	Iloperidone	39.35	−4.35	−6.8	−0.14	648.62	−5.59
	Lamictal	32.04	−4.76	−6.6	−0.30	322.67	−5.36
	Memantine	31.41	−5.60	−6.3	−0.43	79.08	−5.96
	Modafinil	34.20	−5.32	−6.4	−0.28	126.95	−5.99

DAOA-125	Chlorpromazine	—	−4.59	−6.0	−0.22	490.91	−5.71
	Clozapine	43.56	−5.37	−7.1	−0.23	114.85	−5.49
	Galantamine	43.02	−5.17	−6.8	−0.26	162.31	−5.74
	Haloperidol	39.45	−4.96	−8.0	−0.19	231.55	−6.96
	Iloperidone	36.16	−6.22	−6.9	−0.20	27.74	−6.68
	Lamictal	33.15	−3.51	−6.7	−0.22	2.65	−4.06
	Memantine	20.02	−5.50	−6.0	−0.42	92.25	−5.87
	*Modafinil*	*40.54*	−*5.43*	−*6.4*	−*0.29*	*104.74*	−*6.23*

DAOA-126	*Chlorpromazine*	*47.96*	−*5.97*	−*6.3*	−*0.28*	*41.28*	−*6.90*
	Clozapine	43.00	−5.69	−7.4	−0.25	67.96	−5.68
	Galantamine	41.34	−6.22	−6.8	−0.31	27.74	−6.79
	Haloperidol	44.02	−5.91	−7.5	−0.23	46.66	−7.58
	Iloperidone	51.37	−6.31	−7.1	−0.20	32.29	−7.00
	Lamictal	38.52	−4.58	−6.6	−0.29	441.65	−5.16
	Memantine	35.74	−5.29	−6.3	−0.41	132.61	−5.65
	Modafinil	47.26	−5.45	−7.1	−0.29	101.50	−6.05

DAOA-153	*Chlorpromazine*	10.00	−6.06	−6.7	−0.29	36.42	−7.22
	Clozapine	6.64	−3.99	−8.1	−0.17	1.18	−4.96
	Galantamine	20.19	−3.45	−7.1	−0.17	2.96	−3.98
	*Haloperidol*	*27.20*	−*6.57*	−*7.3*	−*0.25*	*15.15*	−*8.58*
	Iloperidone	14.11	−4.55	−7.7	−0.19	1.18	−2.63
	Lamictal	39.37	−4.59	−7.3	−0.29	431.51	−5.20
	Memantine	10.00	−6.17	−6.0	−0.47	30.02	−6.53
	Modafinil	19.25	−5.77	−6.5	−0.30	59.98	−6.36

**Table 3:** The binding residues of selected drugs by utilized docking tools.

Proteins	Drugs	Binding residues (Gold)	Binding residues (AutoDock4)	Binding residues (AutoDock-Vina)
DAOA-82	*Chlorpromazine*	—	Ser-28, Ser-29, Val-31, Gly-32, Phe-35, Tyr-45, Glu-46, Ser-48, Asp-50, Arg-51, Arg-52	Glu-25, Ser-28, Ser-29, Val-31, Gly-32, Phe-35, Tyr-45, Ala-47, Ser-48, Asp-50, Arg-51, Arg-52
	Clozapine	Ala-47, Arg-51, Glu-56, Glu-75. Thr-79	Ser-28, Ser-29, Gly-32, Phe-35, Tyr-45, Arg-51, Arg-52	Ser-28, Val-31, Gly-32, Phe-35, Met-36, Tyr-45, Ser-48, Arg-52
	*Galantamine*	*Arg-52, Trp-59, Trp-60*	*Glu-25, Ser-28, Ser-29, Gly-32, Phe-35, Tyr-45, Ser-48, Arg-51, Arg-52, Pro-54*	*Val-31, Gly-32, Phe-35, Met-36, Tyr-45, Glu-46, Ala-47, Ser-48, Arg-51, Arg-52*
	Haloperidol	Val-14, Pro-20, Trp-59, Thr-60, Asn-62	Ser-28, Ser-29, Val-31, Gly-32, Phe-35, Tyr-45, Glu-46, Ala-47, Ser-48, Arg-51, Arg-52	Glu-25, Ser-28, Ser-29, Val-31, Gly-32, Phe-35, Tyr-45, Ser-48, Arg-51, Arg-52
	Iloperidone	Ala-47, Arg-51, Lys-74, Glu-75	Ser-9, Leu-10, Cys-11, Trp-13, Leu-43, Lys-67, Asp-68, His-73, Ile-76	Ser-28, Ser-29, Val-31, Gly-32, Phe-35, Met-36, Tyr-45, Ala-47, Ser-48, Arg-51, Arg-52
	Lamictal	Glu-69, Ser-70, Cys-71, Asn-72, Ile-76, Lys-74, Glu-75	Glu-25, Ser-28, Val-31, Gly-32, Phe-35, Tyr-45, Ala-47, Ser-48, Arg-51, Arg-52	Ser-28, Ser-29, Val-31, Gly-32, Phe-35, Tyr-45, Glu-46, Ala-47, Ser-48, Arg-51
	Memantine	Tyr-45, Glu-46, Ala-47, Arg-51, Leu-55, Glu-56, Glu-76, Thr-79	Ser-28, Val-31, Gly-32, Phe-35, Tyr-45, Glu-46, Ala-47, Ser-48, Arg-51	Ser-28, Val-31, Gly-32, Phe-35, Tyr-45, Ser-48, Arg-51, Arg-52
	Modafinil	Glu-56, Gln-69, Ser-70, Cys-71, Asn-72, Lys-74, Glu-75, Ile-76	Glu-25, Ser-28, Ser-29, Val-31, Gly-32, Tyr-45, Ser-48, Arg-51, Arg-52	Ser-28, Val-31, Gly-32, Phe-35, Ala-47, Ser-48, Lys-49, Asp-50, Arg-52

DAOA-125	*Chlorpromazine*	—	His-54, Lys-55, Thr-56, Met-101, Cys-104, Ser-113, Cys-114, Asn-115, His-116, Ile-119	His-54, Thr-56, Thr-59, Arg-100, Met-101, Cys-104, Ser-113, Cys-114, Asn-115, His-116, Ile-119
	Clozapine	Ile-5, Trp-8, His-9, Asn-35, Gln-36, Trp-37, Asn-38, Lys-41, Phe-53	His-54, Lys-55, Thr-56, Arg-100, Met-101, Cys-104, Ser-113, Cys-114	Asp-46, Ser-47, Glu-50, Phe-85, Leu-86, Ala-87, Tyr-88
	Galantamine	Lys-55, Thr-56, Thr-59, Leu-67, Ser-71, Arg-100, Met-101, Cys-104, Ile-119	His-54, Lys-55, Thr-56, Met-101, Cys-104, Ser-113, Cys-114, Asn-115, His-116, Ile-119	His-54, Lys-55, Thr-56, Thr-59, Arg-100, Met-101, Cys-114, His-116, Ile-119
	Haloperidol	Val-18, His-54, Lys-55, Thr-59, Arg-94, Pro-97, Met-101, Ile-119	Lys-55, Thr-56, Thr-59, Leu-67, Ser-71, Arg-100, Met-101, Cys-104, Ile-119	Thr-59, Leu-67, Glu-68, Ser-71, Ser-72, Gly-75, Lys-76, Met-79, Met-101, Cys-104, Asn-105, Tyr-106, His-116, Ile-119
	Iloperidone	Leu-9, Val-18, Gly-20, His-54, Lys-55, Thr-56 Met-101, Cys-104, Ile-119, Lys-123	His-54, Lys-55, Thr-56, Thr-59, Leu-67, Pro-97, Arg-100, Met-101, Cys-104, Ser-113, Ile-119	His-54, Lys-55, Thr-56, Thr-59, Pro-97, Met-101, Cys-114, His-116, Ile-119
	Lamictal	His-54, Lys-55, Thr-56, Tyr-83, Met-101, His-116, Ile-119	Arg-100, Met-101, Ser-113, Cys-114, Asn-115, His-116, Ile-119	Leu-25, Gln-29, Thr-33, Asn-35, Asn-38, Met-39, Glu-69, Val-70, His-73
	Memantine	Gly-20, Ser-21, His-54, Thr-56, Ile-119, Ser-121	Lys-55, Thr-59, Leu-67, Ser-71, Val-74, Gly-75, Phe-78, Met-101, Cys-104, Asn-105, Tyr-106	Leu-25, Gln-29, Asn-38, Met-39, Trp-57, Val-70, His-73
	*Modafinil*	*His-54, Lys-55, Thr-56, Thr-59, Tyr-83, Pro-97, Met-101, His116, Ile-119*	*His-54, Lys-55, Thr-56, Thr-59, Met-101, His-116, Ile-119,*	*Lys-55, Thr-59, Leu-67, Ser-71, Met-101, Cys-104, Asn-107, Gln-109, Cys-114, His-116, Ile-119*

DAOA-126	*Chlorpromazine*	*Phe-25, Gly-27, Phe-28, Arg-30, Ser-31, Val-35, Val-55, Thr-56, Glu-59, Arg-63, Leu-70, Gln-74, Leu-88*	*Phe-25, Phe-28, Arg-30, Ser-31, Thr-48, Val-55, Thr-56, Glu-59, Arg-63, Gln-74, Val-85, Leu-88*	*Phe-25, Phe-28, Arg-30, Ser-31, Thr-48, Arg-52, Thr-56, Glu-59, Arg-63, Gln-74, Leu-88, Pro-89*
	Clozapine	Gly-27, Phe-28, Gln-29, Arg-30, Val-55, Thr-56, Glu-59, Arg-63, Gln-74, Gln-78, Val-85, Leu-88	Phe-14, Phe-25, Phe-28, Ser-31, Thr-56, Glu-59, Arg-63, Gln-74, Val-85, Leu-88	Leu-41, Asn-42, Gln-90, Pro-91, Tyr-92, Ala-93, Glu-125
	Galantamine	Val-55, Thr-56, Glu-59, Arg-63, Glu-71, Gln-74, Glu-75, Val-85, Leu-88	Phe-14, Phe-25, Ile-26, Gly-27, Phe-28, Arg-30, Ser-31, Glu-59, Arg-63, Gln-74, Val-85, Leu-88	Leu-5, Leu-41, Asn-42, Tyr-92, Phe-119, Asp-123, Thr-124, Glu-125, Ala-126
	Haloperidol	Phe-25, Gly-27, Phe-28, Arg-30, Ser-31, Thr-48, Arg-52, Thr-56, Glu-59, Arg-63, Gln-74, Leu-88	Phe-25, Ile-26, Phe-28, Arg-30, Ser-31, Thr-48, Arg-52, Thr-56, Glu-59, Arg-63, Gln-74, Val-85, Ser-86, Thr-87, Leu-88, Pro-89	Met-1, Leu-5, Leu-41, Asn-42, Tyr-92, Ala-93, Glu-94, Phe-119, Met-120, Asp-123, Thr-124, Glu-125, Ala-126
	Iloperidone	Phe-25, Gly-27, Phe-28, Gln-29, Arg-30, Thr-48, Arg-52, Val-55, Thr-56, Glu-59, Gln-74, Val-85, Leu-88, Tyr-92, His-95, Ser-96, Ile-99, Phe-115	Gly-27, Phe-28, Arg-30, Thr-56, Glu-59, Arg-63, Gln-74, Gln-78, Val-85, Ser-86, Leu-88, Ile-99	Asn-42, Pro-89, Gln-90, Pro-91, Tyr-92, Ala-93, Phe-119, Asp-123, Thr-124, Glu-125, Ala-126
	Lamictal	Phe-14, Phe-25, Ile-26, Gly-27, Phe-28, Gln-29, Arg-30, Ser-31, Thr-48, Glu-59, Arg-63, Gln-74	Phe-14, Phe-25, Phe-28, Arg-30, Ser-31, Gln-74, Val-85, Leu-88	Leu-5, Leu-41, Asn-42, Tyr-92, Phe-119, Thr-124, Glu-125, Ala-126
	Memantine	Met-1, Gln-29, Leu-41, Asn-42, Tyr-92, Phe-119, Asp-123, Thr-124, Glu-125, Ala-126	Phe-25, Gly-27, Phe-28, Gln-29, Arg-30, Ser-31, Glu-59, Gln-74, Leu-88	Met-1, Leu-5, Gln-29, Leu-41, Asn-42, Tyr-92, Phe-119, Asp-123, Thr-124, Glu-125, Ala-126
	Modafinil	Phe-14, Phe-25, Ile-16, Gly-27, Phe-28, Arg-30, Ser-31, Thr-48, Glu-59, Leu-70, Leu-88	Phe-28, Arg-30, Ser-31, Thr-56, Glu-59, Arg-63, Gln-74, Val-85, Leu-88	Leu-5, Gln-29, Leu-41, Asn-42, Ala-45, Pro-91, Tyr-92, Phe-119, Asp-123, Thr-124, Glu-125, Ala-126

DAOA-153	*Chlorpromazine*	Leu-33, Leu-34, Ser-37, Leu-95, Val-98, Ser-99, Val-102, Tyr-111, Leu-114, Asn-133, Ile-147	Leu-33, Ser-37, Glu-38, Leu-41, Asn-42, Pro-91, Leu-95, Try-130, Lys-138, Asp-139, His-144	Thr-48, Ser-99, Val-102, Gly-103, Lys-104, Leu-114, Glu-117, Ala-118, Ser-119, Gln-124, Ala-152
	Clozapine	Leu-33, Leu-34, Leu-95, Val-98, Ser-99, Val-102, Gly-103, Tyr-111, Leu-114, Tyr-116, Gln-124, Met-129, Trp-130, Asn-133, Ile-147	Leu-34, Leu-95, Val-98, Ser-99, Val-102, Tyr-111, Leu-114, Tyr-116, Gln-124, Met-129, Try-130, Asn-133, Ile-147, Thr-148	Ser-99, Val-102, Gly-103, Leu-114, Tyr-116, Glu-117, Ala-118, Ser-119, Gln-124, Thr-148, Ala-152
	Galantamine	Leu-34, Leu-95, Ser-99, Val-102, Gly-103, Leu-114, Tyr-116, Gln-124, Met-129, Asn-133, Ile-147, Thr-148	Val-98, Ser-99, Val-102, Gly-103, Leu-114, Gln-124, Met-129, Ile-147, Thr-148	Ser-99, Gly-103, Glu-117, Ala-118, Ser-119, Gln-124, Thr-148, Ala-152
	*Haloperidol*	*Leu-33, Leu-34, Ser-37, Ser-40, Leu-41, Ile-44, Leu-95, Val-98, Ser-99, Val-102, Tyr-111, Leu-114, Gln-124, Asn-133, Ile-147, Thr-148*	*Leu-34, Val-98, Ser-99, Val-102, Gly-103, Tyr-111, Leu-114, Ser-119, Asn-133, Ile-147, Thr-148, Ala-152*	*Val-102, Gly-103, Lys-104, Glu-117, Ala-118, Ser-119, Gln-124, Thr-148, Ala-152, Glu-153*
	Iloperidone	Phe-25, Leu-33, Leu-34, Leu-41, Leu-95, Val-98, Ser-99, Val-102, Gly-103, Tyr-111, Leu-114, Glu-117, Gln-124, Asn-133, Ile-147, Thr-148	Leu-33, Leu-34, Ser-37, Glu-38, Ser-40, Leu-41, Leu-95, Val-98, Ser-99, Val-102, Leu-114, Gln-124, Met-129, Ile-147, Thr-148	Ser-99, Gly-103, Lys-104, Leu-114, Tyr-116, Glu-117, Ala-118, Ser-119, Thr-148, Thr-150, Ala-152
	Lamictal	Ser-99, Val-102, Gly-103, Leu-114, Glu-117, Ala-118, Gln-124, Ile-147, Thr-148	Ser-99, Val-102, Gly-103, Leu-114, Tyr-116, Ala-118, Ile-147, Thr-148	Ser-99, Val-102, Gly-103, Leu-114, Glu-117, Ala-118, Ser-119, Gln-124, Ile-147, Thr-148
	Memantine	Leu-33, Leu-34, Leu-95, Val-98, Ser-99, Val-102, Tyr-111, Leu-114, Asn-133, Ile-147	Ser-99, Ser-100, Gly-103, Leu-114, Tyr-116, Ala-118, Gln-124, Ile-147, Thr-148	Ser-99, Ser-100, Leu-114, Tyr-116, Glu-117, Ala-118, Gln-124, Ile-147, Thr-148, Ala-152
	Modafinil	Phe-25, Leu-33, Leu-34, Ser-37, Leu-41, Leu-95, Val-98, Ser-99, Val-102, Tyr-111, Leu-114, Asn-133, Ile-147	Ser-99, Ser-100, Leu-114, Tyr-116, Glu-117, Ala-118, Ser-119, Gln-124, Ile-147, Thr-148, Ser-149	Gly-7, Phe-28, Gln-29, Arg-109, Asn-110, Glu-112, Phe-113, Cys-132

^*∗*^The empty fields are due to the limitation of utilized parameters and software did not run the specific analyses.

**Table 4:** Drug properties of selected drugs.

Ligand properties	Chlorpromazine	Clozapine	Galantamine	Haloperidol	Iloperidone	Lamictal	Memantine	Modafinil
Molecular weight (g/mol)	318.8644	326.8212	273.33	375.8616	426.4794	256.0885	179.3015	273.3518
Hydrogen bond acceptor	2	4	4	3	6	5	1	3
Hydrogen bond donor	0	1	1	1	0	2	1	1
Rotatable bonds	4	1	1	6	8	1	0	5
CLogP	4.61	2.48	1.19	4.3	4.3	1.6	1.98	0.38
Solubility	−4.8	−3.74	−2.66	−4.7	−5.15	−4.36	−2.94	−3.84
Polar surface area	31.78	30.87	41.93	40.54	64.8	90.71	26.02	79.37
Drug-likeness	8.38	8.7	6.2	12.32	6.89	−0.88	−0.8	0.52
Drug score	35%	81%	91%	60%	52%	51%	60%	69%
Logp	4.9594	1.4793	1.1400	4.3635	4.7645	3.1722	3.3944	3.5760
Blood-brain barrier (probability)	0.9795	0.9119	0.9932	0.9465	0.9848	0.9382	0.9823	0.9947
Human intestinal absorption (probability)	0.9757	0.9950	0.9971	1.0000	1.0000	1.0000	0.9939	1.0000
Caco2 permeability (probability)	0.8867	0.5000	0.6717	0.6023	0.5513	0.8867	0.6082	0.5066
CYP450 2D6 inhibitor (probability)	0.9422	0.9090	0.5388	0.9197	0.8860	0.7007	0.8720	0.9117
Carcinogens (probability)	0.9309	0.9315	0.9259	0.8769	0.8699	0.7895	0.7426	0.6665
Acute oral toxicity (probability)	0.7472	0.4670	0.4795	0.7338	0.6524	0.6525	0.7138	0.6472
Aqueous solubility (LogS)	−4.9474	−3.4630	−2.7586	−4.3671	−3.3705	−2.8245	−3.6562	−2.5575
Rat acute toxicity (LD50, mol/kg)	3.3196	2.8265	2.8276	3.4367	2.7862	2.7556	2.3455	2.0926
Fish toxicity (pLC50, mg/L)	1.3654	1.2749	0.5567	1.5595	0.9299	1.3928	1.1422	1.8626
Solvent accessibility surface area (Å^2^)	516.611	501.869	431.455	586.311	652.581	399.087	329.807	444.789
AMES toxicity (probability)	0.9133	0.7280	0.5981	0.9133	0.6314	0.8202	0.6945	0.6562
Honey bee toxicity ( probability)	0.8122	0.8170	0.6512	0.7759	0.7871	0.8834	0.5132	0.6258

**Table 5:** Comparative docking analyses of top 20 novel molecules.

Compounds	AutoDock4 (kcal/mol)	Compounds	AutoDock Vina (kcal/mol)	Compounds	Gold score
DAOA-82					
**SA-1**	**−6.78**	SA-**1**	**−7**	SA-**1**	**43.782**
**SA-3**	**−6.09**	SA-**3**	**−7.5**	SA-**3**	**45.486**
**SA-11**	**−5.78**	SA-106	−6.8	SA-**11**	**45.886**
SA-13	−5.91	SA-7	−6.8	SA-13	44.095
SA-15	−5.94	SA-**11**	**−6.9**	SA-15	42.513
SA-18	−5.78	SA-13	−6.9	SA-18	50.658
SA-28	−5.78	SA-14	−7.2	SA-19	44.565
SA-30	−5.88	SA-18	−6.8	SA-39	45.887
SA-32	−6.46	SA-19	−6.8	SA-32	47.897
SA-33	−5.75	SA-39	−6.9	SA-33	47.920
SA-106	−5.73	SA-47	−7.3	SA-106	43.007
SA-**110**	**−7.17**	SA-49	7.7	SA-**110**	**42.233**
SA-**111**	**−6.19**	SA-**68**	**−7.0**	SA-**111**	**43.603**
SA-**68**	**−6.06**	SA-70	−6.8	SA-**68**	**41.574**
SA-70	−6.67	SA-77	−7.1	SA-70	40.899
SA-14	−5.48	SA-78	−7.8	SA-49	42.105
SA-19	−5.19	SA-**110**	**−6.8**	SA-77	45.099
		SA-**111**	**−6.6**	SA-14	43.535

DAOA-125					
SA-**1**	**−7.87**	SA-**1**	**−8.8**	SA-46	48.981
SA-**3**	**−7.0**	SA-**3**	**−8.6**	SA-5	46.684
SA-**11**	**−6.48**	SA-24	−7.3	SA-24	45.995
SA-63	−6.72	SA-7	−7.7	SA-**3**	**43.184**
SA-15	−6.14	SA-46	−8.4	SA-76	41.242
SA-18	−6.68	SA-49	−7.8	SA-46	48.981
SA-20	−6.97	SA-50	−8.4	SA-50	36.838
SA-61	−6.81	SA-68	−7.4	SA-**1**	**38.631**
SA-**68**	**−6.95**	SA-69	−7.5	SA-59	38.316
SA-69	−7.03	SA-83	−7.5	SA-103	37.983
SA-70	−6.98	SA-84	−7.5	SA-**11**	**25.760**
SA-73	−6.85	SA-**110**	**−7.2**	SA-**68**	**17.444**
SA-**110**	**−7.36**	SA-**111**	**−7.0**	SA-69	20.890
SA-**111**	**−7.55**	SA-14	−7.0	SA-70	34.905
SA-14	−5.75	SA-6	−7.3	SA-73	37.074
SA-6	−6.15	SA-**11**	**−7.0**	SA-**110**	**29.620**
				SA-**111**	**31.280**
				SA-24	45.995

DAOA-126					
SA-**1**	**−8.5**	SA-**1**	**−7.6**	SA-45	62.790
SA-**3**	**−6.91**	SA-**3**	**−8.3**	SA-12	62.569
SA-**11**	**−8.25**	SA-49	−7.5	SA-75	62.168
SA-13	−7.1	SA-7	−7.5	SA-66	62.144
SA-50	−8.04	SA-**11**	**−7.0**	SA-10	61.558
SA-17	−7.02	SA-13	−7.1	SA-25	61.540
SA-2	−7.25	SA-46	−8.0	SA-8	61.379
SA-66	−7.90	SA-50	−8.0	SA-101	60.784
SA-22	−7.15	SA-51	−7.6	SA-96	60.553
SA-46	−7.9	SA-84	−7.0	SA-98	59.892
SA-49	−7.63	SA-2	−6.6	SA-**1**	**50.328**
SA-51	−7.13	SA-**68**	**−7.4**	SA-**3**	**48.548**
SA-**110**	**−7.64**	SA-69	−7.4	SA-**11**	**43.005**
SA-84	−7.47	SA-22	−6.8	SA-13	53.439
SA-**68**	**−7.74**	SA-82	−7.6	SA-**68**	**47.778**
SA-69	−7.85	SA-**111**	**−7.1**	SA-**111**	**50.681**
SA-82	−8.67	SA-**110**	**−6.6**	SA-70	49.059
SA-83	−8.26	SA-66	−7.7	SA-**110**	**48.647**
SA-**111**	**−8.13**	SA-82	−7.6	SA-66	62.144

DAOA-153					
SA-**1**	**−7.21**	SA-**1**	**−8.1**	SA-71	62.740
SA-**3**	**−6.85**	SA-**3**	**−8.4**	SA-49	59.386
SA-**11**	**−6.71**	SA-19	−7.5	SA-12	59.242
SA-22	−6.74	SA-7	−8.0	SA-74	58.613
SA-29	−6.31	SA-46	−8.4	SA-29	55.499
SA-46	−6.73	SA-50	−8.3	SA-101	55.344
SA-**111**	**−6.68**	SA-**68**	**−7.4**	SA-61	55.117
SA-**68**	**−5.68**	SA-69	−7.2	SA-66	54.287
SA-19	−5.89	SA-**110**	**−7.0**	SA-73	53.998
		SA-**111**	**−7.1**	SA-80	53.528
		SA-29	−7.0	SA-**1**	**18.956**
		SA-61	−7.3	SA-**3**	**45.531**
		SA-**11**	**−6.8**	SA-**11**	**41.525**
				SA-29	55.499
				SA-**68**	**40.810**
				SA-**111**	**22.749**
				SA-70	35.919
				SA-**110**	**52.855**
				SA-61	55.117

**Table 6:** Drug properties of selected top 6 novel molecules.

Ligand properties	SA-**1**	SA-**3**	SA-**11**	SA-**68**	SA-**110**	SA-**111**
Molecular weight (g/mol)	434.665	447	377.58	337	365	323
Hydrogen bond acceptor	5	4	6	5	5	5
Hydrogen bond donor	4	3	3	3	3	3
Rotatable bonds	8	9	7	6	6	6
CLogP	2.55	3.62	−0.34	0.76	1.54	0.54
Solubility	−3.71	−4.47	−1.32	−2.06	−2.71	−1.90
Polar surface area	59.56	47.53	70.41	42.57	42.57	42.57
Drug score	47%	30%	89%	59%	63%	91%
Logp	4.6	5.5	2.63	2.8	2.9	2.5
Blood-brain barrier (probability)	0.5737	0.7540	0.5719	0.6063	0.5419	0.6561
Human intestinal absorption (probability)	0.9151	0.9773	0.7924	0.9895	0.9877	0.9853
Caco2 permeability (probability)	0.6103	0.5514	0.6447	0.6103	0.5936	0.6003
CYP450 2D6 inhibitor (probability)	0.6354	0.6787	0.7691	0.6923	0.6956	0.5905
Carcinogens (probability)	0.9018	0.7866	0.7837	0.7750	0.7726	0.7735
Acute oral toxicity (probability)	0.6604	0.6446	0.5971	0.6526	0.6410	0.6475
Aqueous solubility (LogS)	−2.6766	−3.5803	−3.3450	−2.6211	−2.5969	−2.6386
Rat acute toxicity (LD50, mol/kg)	2.4423	2.4860	2.5985	2.6059	2.6503	2.6282
Fish toxicity (pLC50, mg/L)	1.9700	1.6589	1.7700	1.7191	1.6242	1.6695
Solvent accessibility surface area (Å^2^)	593.568	573.21	541.897	556.644	544.502	544.765
AMES toxicity (probability)	0.6291	0.6834	0.6448	0.6112	0.6028	0.5896
Honey bee toxicity ( probability)	0.7875	0.8376	0.6338	0.7393	0.7487	0.7432

**Table 7:** Protein-protein interactions of ligand protein and receptor protein.

Models	Global energy	Attractive VdW	Repulsive VdW	ACE	HB	Ligand protein (DOA)	Receptor protein
DAOA-82	−15.65	−21.29	9.79	5.11	−5.04	Asn-86, Thr-153, Arg-151, Gly-156, Glu-154, Asn-83, His-78, Pro-82, Arg-155, His-20, Glu-21, Arg-22, His-24, Ser-25	Gln-66, Leu-17, Lys-67, Pro-12, Tyr-16, Arg-8, Ser-9, His-5, Thr-77, Leu-43, Phe-42, His-73

DAOA-125	−14.97	−27.04	13.97	7.15	−4.74	Pro-41, Leu-42, Lys-142, Gln-234, Arg-279, Thr-235, Asp-206, Thr-280, Leu-250, Asn-251, Glu-278, Ile-275, Arg-274, His-256, Ile-253, Trp-260, Lys-271, Asn-257, Glu-261	Lys-7, Glu-125, Trp-8, Thr-122, Arg-14, Tyr-17, Val-18, His-16, His116, Pro-97, Arg-100, Arg-94, Gln-96, Asp-93, Ala-90, Ser-91, Lys-92

DAOA-126	−22.87	−31.90	21.72	4.23	−3.91	Thr-269, Arg-120, Pro-268, Glu-267, Leu-266, Arg-265, Met-124, Glu-121, Asp-123, Thr-118, Pro-119, Phe-125, Leu-122, Pro-126, Gly-129	Val-85, Gln-78, Met-72, Ser-86, Gln-74, Arg-75, Tyr-87, Pro-89, Glu-71, Arg-63, Arg-64, Gly-60, Thr-56, Glu-53, Arg-57, Leu-88

DAOA-153	−29.01	−31.15	12.88	1.02	−3.25	Glu-220, Arg-221, Gly-222, Pro-219, Ile-223, His-217, Glu-100, Asn-60, Ala-101, Ile-102, Ser-57, Pro-59, Asp-58, Pro-103, Pro-62, Pro-105, Asp-104, Ser-106	Lys-58, Lys-62, Arg-57, Trp-61, His-65, Pro-83, Gln-78, Gly-68, Glu-71, Arg-75, Arg-79, Tyr-24, Lys-22, Gly-21
