# Supplementary material for: Pharmacoinformatics, Adaptive Evolution, and Elucidation of Six Novel Compounds for Schizophrenia Treatment by Targeting DAOA (G72) Isoforms
Source: Biomed Res Int. 2017 Jan 19;2017:5925714. doi: 10.1155/2017/5925714 (PMC5288522; doi:10.1155/2017/5925714)
Supplement: Supplementary file 1 — Analyses of 10 MB region of selected species and synteny of DAOA. [file 5925714.f1.zip › Supplementary Material/Supp file 1 Model-Evaluation-of-DAOA isoforms_BMRI_1838197.pdf]

Name	ERRAT	F.R.	A.R.	D.R (Outliers)
DAOA-125-M1	63.948	96.2	1.7	2.1
DAOA-125-M2	71.245	94.6	4.2	1.3
DAOA-125-M3	72.103	95	2.5	2.5
DAOA-125-M4	76.623	94.1	4.2	1.7
DAOA-125-M5	80.258	95	3.3	1.7
DAOA-125-M6	71.245	94.1	2.9	2.9
DAOA-125-M7	66.094	96.2	2.9	0.8
DAOA-125-M8	77.253	95	2.5	2.5
DAOA-125-M9	82.833	94.6	3.3	2.1
DAOA-125-M10	69.957	96.7	1.7	1.7
DAOA-125-M11	67.1	93.3	5	1.7
DAOA-125-M12	65.517	93.3	5	1.7
DAOA-125-M13	70.435	93.7	5	1.3
DAOA-125-M14	77.922	94.1	3.3	2.5
DAOA-125-M15	70.815	93.7	4.6	1.7
DAOA-125-M16	79.399	93.7	3.8	2.5
DAOA-125-M17	72.532	94.1	3.8	2.1
DAOA-125-M18	70.815	91.6	6.7	1.7
DAOA-125-M19	69.397	94.1	3.8	2.1
DAOA-125-M20	69.528	92.1	7.1	0.8
DAOA-125-M21	38.182	91.9	4.9	3.3
DAOA-125-M22	66.667	47.2	30.9	22
DAOA-125-M23	76.068	56.9	30.9	12.2
DAOA-125-M24	79.487	52	23.5	15.4
DAOA-125-M25	62.931	61.8	25.2	13
DAOA-125-M26	60.684	52	25.2	22.8

Name	ERRAT	F.R.	A.R.	D.R (Outliers)
DAOA-82-M1	58.333	91.2	7.5	1
DAOA-82-M2	39.062	96.2	3.8	0
DAOA-82-M3	35.616	90	6.2	3.8
DAOA-82-M4	45.07	92.5	5	2.5
DAOA-82-M5	58.824	92.5	3.8	3.8
DAOA-82-M6	39.13	90	6.2	3.8
DAOA-82-M7	40.845	93.8	3.8	2.5
DAOA-82-M8	40	87.5	7.5	5
DAOA-82-M9	35.714	92.5	5	2.5
DAOA-82-M10	51.562	93.8	5	1.2
DAOA-82-M11	55.882	88.8	11.2	0
DAOA-82-M12	28.767	92.5	6.2	1.2
DAOA-82-M13	27.397	91.2	6.2	2.5
DAOA-82-M14	46.479	95	3.8	1.2
DAOA-82-M15	30.137	93.8	3.8	2.5
DAOA-82-M16	52.113	95	0	5
DAOA-82-M17	33.333	95	3.8	1.2
DAOA-82-M18	25.373	90	5	5
DAOA-82-M19	43.836	88	6.2	5
DAOA-82-M20	52.055	93.8	1.2	5
DAOA-82-M21	42.254	92.5	7.5	0
DAOA-82-M22	85.135	48.8	25	26.2
DAOA-82-M23	58.108	60	26.2	13.8
DAOA-82-M24	64.865	40	32.5	27.5
DAOA-82-M25	72.973	60	21.2	18.8
DAOA-82-M26	68.919	51.2	33.8	15
DAOA-82-M27	37.838	80	12.5	7.5

Name	ERRAT	F.R.	A.R.	D.R (Outliers)
DAOA-126-M1	52.991	88.7	8.1	3.2
DAOA-126-M2	36.207	92.7	6.5	0.8
DAOA-126-M3	42.056	91.9	4.8	3.2
DAOA-126-M4	24.324	88.7	9.7	1.6
DAOA-126-M5	24.348	88.7	6.5	4.8
DAOA-126-M6	30.172	87.1	10.5	2.4
DAOA-126-M7	30.909	84.7	10.5	4.8
DAOA-126-M8	29.565	86.3	11.3	2.4
DAOA-126-M9	40.351	91.1	6.5	2.4
DAOA-126-M10	35.345	86.3	9.7	4
DAOA-126-M11	30.508	62.9	20.2	16.9
DAOA-126-M12	80.508	62.1	20.2	17.7
DAOA-126-M13	53.39	54	27.4	18.5
DAOA-126-M14	61.017	72.6	16.9	10.5
DAOA-126-M15	83.898	79	12.1	8.9
DAOA-126-M16	28.814	85.5	7.3	7.3
DAOA-126-M17	25.862	87.1	10.5	2.4

Name	ERRAT	F.R.	A.R.	D.R (Outliers)
DAOA-153-M1	43.75	88.1	9.3	2.6
DAOA-153-M2	32.624	92.7	4	3.3
DAOA-153-M3	18.705	91.4	6.6	2
DAOA-153-M4	28.169	92.7	3.3	4
DAOA-153-M5	23.611	83.4	10.6	6
DAOA-153-M6	40.845	90.7	4.6	4.6
DAOA-153-M7	38.462	90.1	7.9	2
DAOA-153-M8	30.986	90.1	6	4
DAOA-153-M9	25.874	90.1	6	4
DAOA-153-M10	21.127	87.4	7.9	4.6
DAOA-153-M11	88.966	83.4	11.3	5.3
DAOA-153-M12	90.345	65.6	29.8	4.6
DAOA-153-M13	97.931	67.5	19.2	13.2
DAOA-153-M14	87.586	74.2	15.2	10.6
DAOA-153-M15	27.143	92.7	4.6	2.6
DAOA-153-M16	79.07	93.4	4	2.6
