# Supplementary material for: Pharmacoinformatics, Adaptive Evolution, and Elucidation of Six Novel Compounds for Schizophrenia Treatment by Targeting DAOA (G72) Isoforms
Source: Biomed Res Int. 2017 Jan 19;2017:5925714. doi: 10.1155/2017/5925714 (PMC5288522; doi:10.1155/2017/5925714)
Supplement: Supplementary file 1 — Analyses of 10 MB region of selected species and synteny of DAOA. [file 5925714.f1.zip › Supplementary Material/Supp File 2Top 10 binding pockets by SiteHound.pdf]

Top ten predicted binding pockets through SiteHound

DAOA82

Rank	Energy (kcal/mol)	Energy Range	Volume (Å)	Center x	Center y	Center z	Residues
1	-718.72	(-17.14, -8.93)	63.00	24.955	20.339	27.888	Glu-25, Ser-28, Ser-29, Val-31, Gly-32, Phe-35, Tyr-45, Glu-46, Ala-47, Ser-48, Asp-50, Arg-51, Arg-52
2	-538.67	(-15.21, -8.91)	51.00	26.972	39.293	28.491	Arg-8, Ser-9, Leu-10, Cys-11, Pro-12, Trp-13, Ser-15, Tyr-16, Leu-17, Tyr-63, Asn-64, Gln-66, Lys-67, Asp-68
3	-309.49	(-14.45, -9.02)	28.00	18.151	25.082	35.550	Tyr-21, Leu-24, Glu-25, Arg-52 Gln-53 Pro-54, Arg-57, Trp-59, Thr-60
4	-281.54	(-15.14, -8.97)	25.00	34.866	33.473	30.34	Ser-9, Leu-10, Leu-43, Lys-67, Asp-68, Cys-71, Asn-72, His-73, Lys-74, Ile-76, Thr-77
5	-277.95	(-16.79, -9.03)	24.00	21.289	31.801	19.518	Gln-7, Arg-8, Pro-12, Tyr-16, Leu-17, Glu-23, Glu-26, Val-27, His-30, Lys-33
6	-215.09	(-16.07, -8.92)	19.00	16.884	35.276	34.631	Val-14, Ser-15, Pro-18, Pro-20, Trp-59, Thr-60, Asn-62
7	-204.36	(-16.81, -8.93)	18.00	33.796	15.296	23.977	Phe-35, Met-36, Arg-38, Glu-41, Lys-80,Glu-82
8	-186.51	(-13.27, -8.92)	19.00	33.680	26.769	37.55	Tyr-45, Glu-46, Ala-47, Arg-51, Leu-55, Cys-71, Asn-72, Lys-74, Glu-75, Ile-76, Thr-79
9	-153.11	(-14.61, -9.04)	14.00	34.888	38.377	38.810	Gln-65, Gln-66, Lys-67, Asp-68, Gln-69, Asn-72, His-73, Lys-74
10	-136.84	(-11.70, -8.95)	14.00	41.486	27.992	33.142	His-73, Lys-74, Glu-75, Thr-77, Ser-78, Thr-79

DAOA125

Rank	Energy (kcal/mol)	Energy Range	Volume (Å)	Center x	Center y	Center z	Residues
1	-1209.46	(-20.79, -8.90)	94.00	40.579	32.885	30.109	Lys-55, Thr-59, Leu-67, Glu-68, Ser-71, Ser-72, Gly-75, Lys-76, Phe-78, Met-79, Glu-84, Met-101, Trp-102, Cys-104, Asn-105, Tyr-106, Asn-107
2	-918.81	(-13.37, -8.92)	88.00	36.265	28.999	22.485	Phe-53, His-54,Lys-55, Thr-56, Thr-59, Leu-67, Tyr-83, Pro-97, Arg-100, Met-101, Cys-104, Ser-113,Cys-114, Asn-115, His-116, Ile-119
3	-734.39	(-18.02, -8.90)	66.00	20.845	37.491	35.600	Arg-2, Ile-5, Trp-6, His-9, Asn-35, Gln-36, Trp-37, Asn-38, Lys-41, Phe-53
4	-536.17	(-15.86, -8.97)	47.00	30.044	44.585	31.348	Leu-25, Gln-29, Thr-33, Ser-34, Asn-35, Asn-38, Met-39, Gly-40, Trp-57, Glu-69, Val-70, His-73
5	-527.45	(-15.92, -8.92)	49.00	27.654	16.440	35.958	His-16, Leu-45, Asp-46, Ser-47, Gly-48, Asp-49, Glu-50, Phe-85, Leu-86, Ala-87, Tyr-88, Glu-89, Ala-90, Arg-94
6	-507.06	(-17.42, -9.20)	43.00	37.345	37.511	19.112	Thr-59, Glu-60, Thr-61, Gly-62, Pro-64, Tyr-65, Arg-66, Leu-67, Cys-114, His-116, Lys-117, Glu-118, Ile-119, Thr-120
7	-406.91	(-20.38, -9.07)	33.00	43.928	33.062	22.207	Leu-67, Cys-104, Tyr-106, Asn-107, Gln-108, Gln-109, Lys-110, Asp-111, Ser-113, Cys-114
8	-380.91	(-14.14, -8.93)	36.00	31.655	21.853	40.301	Leu-45, Asp-46, Ser-47, Arg-81, Asn-82, Tyr-83, Glu-84, Phe-85, Leu-86
9	-256.93	(-13.58, -9.14)	24.00	13.565	26.632	27.938	Trp-6, Lys-7, Trp-8, Arg-10, Gly-11, Arg-14, Tyr-17
10	-225.83	(-13.52, -8.94)	21.00	28.653	28.604	17.321	Ser-21, His-54, Thr-56, Glu118, Ile-119, Thr-120, Ser-121, Ser-121, Thr-122, Lys-123

DAOA126

Rank	Energy (kcal/mol)	Energy Range	Volume (Å)	Center x	Center y	Center z	Residues
1	-972.29	(-17.22, -8.90)	83.00	31.864	27.764	26.947	Phe-14, Phe-25, Gly-27, Phe-28, Gln-29, Arg-30, Ser-31, Thr-48, Arg-52, Val-55, Thr-56, Glu-59, Arg-63, Leu-70, Gln-74, Leu-77, Gln-78, Val-85, Ser-86, Tyr-87, Leu-88, Pro-89, His-95, Ile-99
2	-791.47	(-13.80, -8.90)	73.00	28.529	18.190	39.681	Met-1, Leu-5, Gln-29, Ser-40, Leu-41, Asn-42, Ala-45, Lys-46, Pro-91, Tyr-92, Ala-93, Phe-119, Asp-123, Thr-124, Glu-125, Ala-126
3	-290.84	(-15.37, -8.97)	25.00	20.168	27.371	21.653	Arg-17, Ser-31, Ile-34, Ser-35, Lys-62, Glu-66, Leu-70
4	-267.64	(-13.41, -9.06)	26.00	15.282	33.776	19.135	Arg-15, Ser-16, Arg-17, Tyr-18, Thr-19, His-65, Glu-66
5	-236.05	(-18.30, -8.99)	19.00	33.640	19.598	33.901	Gln-29, Ala-45, Lys-46, Thr-48, Glu-49, Leu-88, Pro-89, Gln-90, Pro-91, Tyr-92
6	-221.83	(-21.21, -8.94)	16.00	22.627	20.561	42.396	Met-1, Glu-3, Lys-4, Leu-5, His-118, Phe-119, Gly-122, Asp-123, Thr-124
7	-198.99	(-12.14, -8.95)	20.00	27.281	41.152	43.029	Leu-100, Asn-101, Cys-106, Lys-109, Arg-110, Gln-113
8	-192.81	(-17.97, -9.69)	13.00	28.606	26.326	35.692	Leu-5, Met-6, Gly-27, Phe-28, Gln-29, Leu-33, Leu-88, Tyr-92, His-95, Ser-96, Phe-115
9	-191.27	(-13.10, -8.92)	19.00	31.601	44.119	33.995	Tyr-24, His-76, Arg-79, Ser-80, Gly-102, Asn-103, Leu-104, His-105, Cys-106, Lys-109
10	-179.79	(-14.56, -9.30)	16.00	28.730	35.321	14.524	Arg-63, Arg-64, Glu-66, Asp-67, Gly-68, Glu-71, Met-72, Arg-75

DAOA153

Rank	Energy (kcal/mol)	Energy Range	Volume (Å)	Center x	Center y	Center z	Residues
1	-1073.98	(-17.67, -9.00)	89.00	21.365	23.067	29.915	Ser-99, Ser-100, Val-102, Gly-103, Leu-114, Tyr-116, Glu-117, Ala-118, Ser-119, Lys-120, Gln-124, Met-129, Ile-147, Thr-148, Ser-149, Ala-152
2	-623.73	(-18.88, -8.98)	52.00	31.007	20.691	21.272	Arg-79, Ser-80, Leu-81, Cys-82, Trp-84, Val-85, Tyr-87, Glu-97, Ser-100, His-101, Lys-104, Lys-151, Glu-153
3	-558.40	(-14.33, -8.91)	53.00	21.403	17.801	41.041	Leu-2, Glu-3, Leu-5, Met-6, Asp-9, Phe-106, Met-107, Asn-110, Phe-113, Ala-115, Tyr-116, Ala-118, Asp-121, Arg-122, Arg-123
4	-518.09	(-15.81, -9.15)	46.00	40.769	40.630	32.475	Lys-36, Glu-38, Asn-39, Ser-40, Asn-42, Ser-43, Lys-46, Arg-63, Arg-64, Glu-66, Asp-67, Leu-70
5	-513.77	(-14.72, -8.95)	49.00	28.183	27.492	45.292	Glu-3, Lys-4, Met-6, Gly-7, Ala-8, Leu-11, Gly-27, Phe-28, Gln-29, Ser-31, Arg-109, Asn-110, Glu-112, Phe-113
6	-433.85	(-15.53, -8.96)	40.00	41.151	27.331	20.288	Thr-48, Glu-49, Glu-50, Gly-51, Arg-52, Val-55, Leu-77, Gln-78, Leu-81, Pro-83, Trp-84, Val-85, Leu-88
7	-333.57	(-14.53, -8.93)	31.00	25.133	38.155	27.925	Ser-37, Glu-38, Leu-41, Pro-91, Tyr-92, Leu-95, Trp-130, Lys-138, Asp-139, Cys-142, His-144
8	-319.07	(-18.86, -8.90)	28.00	46.581	29.296	38.183	Tyr-24, Trp-61, Arg-64, His-65, Gly-68, Tyr-69, Glu-71
9	-319.07	(-18.86, -8.90)	28.00	46.581	29.296	38.183	Arg-17, Gly-103, Lys-104, Val-105, Phe-106, Ala-118, Ser-119, Ala-152, Glu-153
