# Supplementary material for: Pharmacoinformatics, Adaptive Evolution, and Elucidation of Six Novel Compounds for Schizophrenia Treatment by Targeting DAOA (G72) Isoforms
Source: Biomed Res Int. 2017 Jan 19;2017:5925714. doi: 10.1155/2017/5925714 (PMC5288522; doi:10.1155/2017/5925714)
Supplement: Supplementary file 1 — Analyses of 10 MB region of selected species and synteny of DAOA. [file 5925714.f1.zip › Supplementary Material/Supp File 3 Protein-Protein Interaction Analysis of top ten complexes.pdf]

Protein-Protein Interaction Analysis of top ten complexes

DAOA-82

Models	Global Energy	Attractive VdW	Repulsive VdW	ACE	HB	DOA	DAOA
Soln-1	-15.65	-21.29	9.79	5.11	-5.04	Asn-86, Thr-153, Arg-151, Gly-156, Glu-154, Asn-83, His-78, Pro-82, Arg-155, His-20, Glu-21, Arg-22, His-24, Ser-25	Gln-66, Leu-17, Lys-67, Pro-12, Tyr-16, Arg-8, Ser-9, His-5, Thr-77, Leu-43, Phe-42, His-73
Soln-2	-4.08	-29.15	26.73	9.09	-1.39	Gly-277, Ile-276, Leu-42, Phe-39, Pro-41, Thr-40, Lys-142, Asn-143, Gln-146, Glu-150, Thr-233, Gln-234, Asn-86, Glu-85, Pro-82, Phe-159	Gln-66, Lys-67, His-73, Leu-17, Lys-11, Ser-9, Thr-77, Arg-8, Leu-43, His-5, Arg-4, Ala-2, Met-1
Soln-3	8.46	-6.57	1.95	2.10	-1.37	Asn-252, Ile-253, Gln-201, Gln-254, Asp-255, Gln-243, Asn-257, Ile-241, Phe-242, Thr-258, Glu-261, Asn-225, Gly-262, Arg-265, Arg-221, Asp-218, Glu-220	Lys-74, Glu-75, His-73, Thr-79, Ile-76, Lys-80, Thr-79, Ile-76, Lys-80, Thr-77, Ser-78, Ala-81, Glu-82, Leu-43, His-5
Soln-4	8.61	-2.10	1.16	-0.97	0.00	Arg-265, Glu-261, Asn-257, Trp-260	Ser-29, Lys-33, Ala-37, Met-36
Soln-5	10.61	-23.11	16.85	2.49	-3.03	Glu-100, Glu-220, Pro-219, Ser-57, Phe-98, Arg-22, Gly-222, His-217, Asn-60, Pro-287, Arg-286, Gln-196, Leu-244, Val-285	Glu-26, Ser-20, Gly-32, Tyr-40, Phe-35, Ala-37, Ser-48, Met-36, Asp-50, Lys-80, Asn-39, Glu-82, Arg-38
Soln-6	11.92	-30.37	33.90	11.96	-2.24	Thr-235, Lys-204, Ile-276, Gly-277, Glu-278, Thr-233, Gln-234, Arg-279, Glu-85, Asn-143, Lys-142, Asn-86, Gln-146, Leu-42, Glu-150, Thr-149, Thr-153	Asn-64, Asn-62, Gln-65, Gln-66, Pro-18, Gln-19, Leu-17, Trp-13, Lys-67, Pro-12, Cys-11, His-73, Arg-8, Arg-4
Soln-7	13.37	-6.27	4.19	-0.67	0.00	Ser-81, His-80, Ser-77, Leu-76, Asp-73, Leu-112	Leu-17, Gln-19, Tyr-16, Glu-23, Ala-22, Glu-26
Soln-8	17.58	-5.24	1.00	6.31	-0.49	Lys-211, Gln-234, Glu-85	Thr-60, Arg-57, Arg-51, Ala-47, Ser-48, Asp-50, Lys-49
Soln-9	28.26	-21.36	8.91	11.22	-1.70	Phe-159, Thr-149, Gln-146, Asn-143, Phe-39, Gln-161, Thr-40, Lys-142, Pro-41, Gln-234, Lys-204, Asp-206	Leu-17, Gln-19, Tyr-16, Cys-11, Arg-8, Glu-23, Gln-7, Arg-4, Met-1
Soln-10	580.16	-28.70	680.93	18.34	-2.98	Pro-62, Gln-63, Asn-61, Asn-60, Gln-288, Pro-287, Arg-286, Leu-194, Ile-289, Glu-292, Leu-291, Arg-293, Gln-295, Glu-336, Leu-329, Arg-332	Gln-65, Asn-62, Ser-70, Cys-61, Pro-20, Thr-60, Trp-59, Ala-22, Thr-21, Arg-57, Glu-56, Gln-53

DAOA-125

Models	Global Energy	Attractive VdW	Repulsive VdW	ACE	HB	DOA	DAOA
Soln-1	-14.97	-27.04	13.97	7.15	-4.74	Pro-41, Leu-42, Lys-142, Gln-234, Arg-279, Thr-235, Asp-206, Thr-280, Leu-250, Asn-251, Glu-278, Ile-275, Arg-274, His-256, Ile-253, Trp-260, Lys-271, Asn-257, Glu-261	Lys-7, Glu-125, Trp-8, Thr-122, Arg-14, Tyr-17, Val-18, His-16, His116, Pro-97, Arg-100, Arg-94, Gln-96, Asp-93, Ala-90, Ser-91, Lys-92
Soln-2	6.33	-24.48	33.98	3.36	-2.62	Gln-254, Thr-258, Gly-262, Pro-126, Asp-127, Tyr-128, Pro-227, His-99, Arg-221, Glu-220, Pro-219, Gly-222, Gln-243, Phe-242, Leu-244	Thr-120, Glu-118, Glu-125, Thr-122, His-116, Lys-123, Lys-7, Gly-20, His-54, Leu-19, Pro-97, Val-18, Tyr-17
Soln-3	7.68	-18.80	16.51	7.17	-2.92	Asp-58, Asn-60, Pro-59, Ser-57, Gln-288, Pro-287, Tyr-55, Tyr-314, Arg-286, Leu-194, Pro-219, Glu-220, Gln-196, Leu-244	Lys-117, Glu-118, Lys-110, Asp-111, His-116, Gln-112, Ser-113, Gln-109, Arg-100, Thr-122
Soln-4	11.35	-12.38	2.76	2.72	-0.60	Gln-234, Thr-233, Gly-232, Pro-208, Asp-206, Lys-211, Trp-209, Arg-274, Asn-272, Pro-268, Met-124	Ile-119, Ser-121, His-116, Lys-123, Thr-122, Val-18, Tyr-17, Ser-91, Lys-92
Soln-5	17.55	-33.02	14.48	19.10	-2.97	Arg-293, Leu-320, Glu-294, Glu-292, Glu-325, Gln-63, Asp-66, Trp-67, Asn-61, Arg-290, Gln-190, Gln-288, Asn-60, Arg-286, Asp-58, Ser-57	Ile-119, Lys-117, Glu-118, Lys-123, Thr-122, His-116, Asn-115, Gln-112, Arg-100, Pro-97, Val-18, Gln-96, Asp-93, Ser-91, Lys-92
Soln-6	19.13	-26.66	12.33	13.53	-2.98	Arg-332, Glu-336, Gln-63, Arg-293, Asn-61, Glu-292, Gln-295,Asn-302, Asn-60, Gln-288, Arg-290, Arg-286, Glu-294, Arg-297, Gln-190, Leu-194, Pro-193	Gly-32, Ala-31, Arg-30, Met-1, Thr-3, Trp-6, Lys-7, Glu-125, Ala-124, Thr-120, Ser-121, Thr-122, Glu-118, Tyr-17
Soln-7	22.44	-6.07	1.31	5.70	-0.64	Lys-338, Lys-337, Glu-335, Glu-336, Pro-300, Ser-301, Asn-302, Ile-333 Glu-304, Arg-332, Arg-297, Arg-293, Leu-329, Glu-325, Glu-292, Gln-190, Arg-290, Arg-191, Asp-192, Arg-286, Pro-193, Leu-194	Glu-118, Ile-119, His-116, Ser-121, Ala-124, Thr-122, Glu-125, Lys-123, Ser-21, Trp-8, Tyr-17, Val-18, Asp-93, Arg-94, Lys-92, Ser-91, Ala-90, Glu-89
Soln-8	27.53	-15.22	5.55	10.56	-2.89	Gln-234, Asn-143, Lys-204, Ile-275, Ile-276, Lys-142, Gln-146, Gly-277, Glu-278, Arg-279, Pro-41, Leu-42	Thr-33, Ser-34, Asn-35, Gln-36, Met-1, Arg-2, Thr-3, His-9, Thr-44, Lys-41, Trp-37, Asn-38
Soln-9	41.57	-12.86	36.42	10.95	-0.43	Gln-234, Thr-235, Pro-208, Trp-209, Arg-274, Asn-272	Gln-36, Arg-2, Lys-41, Trp-6, His-9, Thr-44, Arg-10, Tyr-13
Soln-10	43.59	-33.05	63.67	6.83	-3.27	Ser-301, Lys-338, Lys-337, Leu-339, Glu-336, Asp-176, Glu-335, Ser-340, Arg-332, Met-1, Gln-28, Pro-29, Leu-27, Val-26, Ser-25	Glu-118, Ile-119, His-116, Ser-121, Thr-56, Thr-122, Glu-125, Lys-123, His-54, Pro-97, Trp-8, Leu-19, Val-18, Tyr-17, Asp-93, Lys-92

DAOA-126

Models	Global Energy	Attractive VdW	Repulsive VdW	ACE	HB	DOA	DAOA
Soln-1	-22.87	-31.90	21.72	4.23	-3.91	Thr-269, Arg-120, Pro-268, Glu-267, Leu-266, Arg-265, Met-124, Glu-121, Asp-123, Thr-118, Pro-119, Phe-125, Leu-122, Pro-126, Gly-129	Val-85, Gln-78, Met-72, Ser-86, Gln-74, Arg-75, Tyr-87, Pro-89, Glu-71, Arg-63, Arg-64, Gly-60, Thr-56, Glu-53, Arg-57, Leu-88
Soln-2	-19.15	-31.46	11.45	2.15	-1.80	Pro-193, Leu-194, Leu-244, Pro-287, Phe-242, Asn-60, Gly-222, Ile-223, Arg-221, Glu-220, Pro-219, Ser-57, Asp-58, Ile-102, Glu-100, Leu-244, Pro-103, Ala-101	Arg-110, Thr-19, Asp-9, Gln-12, Arg-17, Arg-15, Leu-11, His-118, Lys-62, Leu-34, Glu-3, Ser-37, Lys-36, Glu-38, Thr-19, Arg-15, Gly-7
Soln-3	-12.09	-32.47	10.98	8.25	-3.21	Arg-115, Glu-121, Gly-113, Thr-135, Ser-93, His-212, Glu-267, Lys-211, Thr-269, Met-210, Trp-209, Leu-91, His-80, Val-79, Phe-90, Gly-232, Leu-89, Thr-233, Glu-85	Gln-12, Arg-17, Arg-15, Leu-11, Lys-62, Trp-61, Ser-35, Ile-32, Glu-3, Leu-2, Ser-37, Lys-36, Glu-38, Asn-39
Soln-4	0.44	-11.63	6.38	4.11	-2.72	Gly-277, His-256, Glu-278, Ile-253, Arg-273, Gln-254, Asn-251, Asn-252, Pro-41, Thr-40, Thr-280, Leu-42, Leu-250, Thr-43, Glu-249, Phe-39, Arg-38, Ser-248, Gln-161, Asp-37, Ala-36, Arg-162, Lys-163	Tyr-18, Thr-19, Ser-16, Arg-17, Glu-66, Gln-12, His-65, Asp-9, Arg-15, Ala-8, Ser-10, Leu-11, Gly-7, Lys-62, Leu-34, Met-6, His-118, Ser-35, Ser-37, Lys-4, Glu-3, Leu-2, Met-1
Soln-5	8.00	-14.12	5.88	6.26	-2.01	Ser-25, His-24, Glu-21, Arg-155, Glu-154, Arg-151, His-78, Asn-83, Pro-82	His-107, Arg-110, Ile-111, Leu-16, Asp-9, Gln-12, Arg-15, Ala-8, Leu-11, Gly-7, Leu-34, Ser-35, Ser-37, Lys-36, Glu-38
Soln-6	8.40	-29.99	17.28	10.52	-3.59	Leu-339, Lys-338, Glu-336, Lys-337, Met-1, Asp-176, Glu-304, Ala-175, Gly-174, Gln-295, Ser-301, Pro-300, Arg-297, Arg-172	Tyr-69, His-76, Arg-79, Met-72, Ala-73, Arg-75, Gln-78, Lys-32, Gln-74, Val-85, Po-83, Ser-86, Leu-88, Tyr-87, Thr-56
Soln-7	12.88	-21.53	19.42	8.61	-1.03	Arg-151, Thr-153, Glu-150, Asn-86, Trp-147, Glu-85, Gln-146, Asn-143, Lys-142, Gln-234, Leu-42, Lys-204, Ile-276	Arg-79, Leu-81, Arg-75, Lys-82, Gln-78, Pro-83, Trp-84, Ser-86, Tyr-81, Gln-90, Arg-52, Glu-53
Soln-8	15.38	-29.44	14.73	16.26	-2.78	Glu-336, Arg-332, Leu-329, Glu-325, Arg-293, Asn-302, Gln-295, Gln-63, Glu-292, Glu-294, Asn-61, Arg-297, Arg-290, Gln-288, Asn-60, Gln-190, Arg-286, Asp-192, Leu-194	Cys-106, His-105, His-107, Arg-110, Lys-22, Ile-111, Ile-114, Thr-19, Leu-13, Asp-9, Glu-121, His-118, Ala-3, Gly-7, Gln-12, Ser-16, Arg-17, Thr-18
Soln-9	17.57	-34.85	23.15	11.98	-6.04	His-80, Thr-135, Ser-93, Leu-91, His-212, Lys-211, Phe-90, Trp-209, Pro-231, Pro-208, Glu-85, Gly-232, Thr-233, Gln-234, Thr-235, Asp-206	Tyr-24, Tyr-69, Asp-67, Gly-68, His-76, Met-72, Gln-74, Arg-63, Glu-71, Arg-64, Arg-75, Gln-78, Cys-82, Gly-60, Thr-56, Arg-57, Arg-79
Soln-10	21.54	-23.00	11.32	12.03	-1.55	Gln-28, Asp-31, Leu-339, Lys-33, Lys-337, Asp-176, Arg-2	Gln-74, Gln-78, Ser-86, Tyr-87, Pro-89, Thr-56, Arg-52, Arg-57, Glu-53

DAOA-153

Models	Global Energy	Attractive VdW	Repulsive VdW	ACE	HB	DOA	DAOA
Soln-1	-29.01	-31.15	12.88	1.02	-3.25	Glu-220, Arg-221, Gly-222, Pro-219, Ile-223, His-217, Glu-100, Asn-60, Ala-101, Ile-102, Ser-57, Pro-59, Asp-58, Pro-103, Pro-62, Pro-105, Asp-104, Ser-106	Lys-58, Lys-62, Arg-57, Trp-61, His-65, Pro-83, Gln-78, Gly-68, Glu-71, Arg-75, Arg-79, Tyr-24, Lys-22, Gly-21
Soln-2	-5.84	-30.04	16.20	3.62	-2.37	Asp-206, Lys-142, Ile-276, Arg-274, Ile-275, Gly-277, Arg-279, Pro-41, Leu-42, Glu-278, Asn-251, Glu-249, leu-250, Ser-243, Trp-247, Lys-163, Ile-253	Lys-4, Leu-5, Ala-8, Gln-29, Phe-28, Leu-11, Gln-12, Ile-26, Arg-15, Arg-30, Tyr-69, Asp-67, Gly-68, Leu-20, Tyr-24, Glu-66, His-65, Lys-62, Gly-21
Soln-3	-5.58	-14.23	6.09	3.79	-0.99	Pro-62, Asp-58, Asp-104, Pro-103, Pro-287, Leu-194, Ile-223, Glu-220, Gly-222, Leu-244	His-65, Trp-61, Arg-57, Thr-54, Tyr-24, Arg-15, Arg-79, Leu-20, Lys-22, Thr-19
Soln-4	-0.24	-17.67	6.49	7.64	-3.73	Glu-220, Pro-219, Glu-100, Ala-101, Asn-60, Ile-102, Pro-103, Asp-58, Asp-104, Ser-106, Lys-108, Asp-109, Pro-62	Lys-58, Arg-64, His-65, Arg-57, Trp-61, Pro-83, Glu-71, Arg-75, Arg-79, Tyr-24, Lys-22, Gly-21, Leu-20,Thr-19
Soln-5	0.38	-8.93	1.87	1.22	-0.69	Glu-100, His-99, Pro-219, Glu-220, Asp-218, Ile-223, Gly-222, Arg-221, Phe-242, Leu-244, Gly-245, Asn-257, Gln-254	Arg-52, Trp-84, Val-85, Pro-83, Thr-54, Val-55, Arg-57, Gln-78, Trp-61, Lys-22, Thr-19
Soln-6	6.49	-4.18	2.40	-0.66	-1.55	His-80, Pro-82,Val-79, Ala-84, Lys-211, Thr-233, Met-210, Pro-208, Trp-209, Gln-234, Ala-207, Val-236, Thr-269, Asp-206	Glu-66, His-65, Trp-61, Ile-26, Phe-25, Tyr-24, Leu-11, Arg-15, Tyr-24, Leu-13, Gln-12, Tyr-18, Leu-20, Ser-16, Gly-21, Thr-19, Ala-8
Soln-7	9.33	-35.97	13.72	22.30	-3.09	His-80, Ser-81, Pro-82, Ala-84, Phe-90, Arg-151, Asn-85, Glu-85, Glu-150, Lys-211, Gly-232, Thr-233, Gln-146, Lys-142, Gln-234, Thr-40, Pro-208, Pro-41, Asp-206	Gln-136, Asn-135, Arg-128, Gln-29, Cys-132, Pro-125, Phe-113, Gln-124, Ala-115, Arg-122, Arg-123, Glu-3, Lys-4
Soln-8	10.54	-26.12	13.31	13.19	-3.77	Ile-253, Leu-250, Asn-251, His-256, Glu-249,Thr-280, Glu-278,Arg-279, Ile-275, Arg-274, Ile-276, Arg-38, Leu-42, Pro-41, Phe-39, Thr-40, Lys-142, Gln-146, Thr-149	Ser-141, Lys-138, Lys-142, Glu-38, Lys-36, Tyr-92, Asn-42, Pro-91, Lys-46, Glu-49, Gly-51, Glu-59, Lys-62, Arg-63, Glu-66
Soln-9	13.88	-9.46	10.01	2.60	-2.26	Val-79, His-80, Phe-90, Thr-233, Gln-234, Leu-91, Leu-112, Gly-232, Thr-235, Asp-206, Pro-208, Lys-211, Trp-209	Ser-35, Gln-29, Arg-30, Ser-37, Phe-28, Glu-66, Lys-62, His-68, Gly-68, Tyr-69, Trp-61, Tyr-24
Soln-10	894.38	-52.30	1157.00	9.28	-5.94	Lys-158, Glu-150, Thr-40, Gln-146, Lys-142	Asn-135, Glu-3, Lys-4
