# Supplementary material for: Pharmacoinformatics, Adaptive Evolution, and Elucidation of Six Novel Compounds for Schizophrenia Treatment by Targeting DAOA (G72) Isoforms
Source: Biomed Res Int. 2017 Jan 19;2017:5925714. doi: 10.1155/2017/5925714 (PMC5288522; doi:10.1155/2017/5925714)
Supplement: Supplementary file 1 — Analyses of 10 MB region of selected species and synteny of DAOA. [file 5925714.f1.zip › Supplementary Material/Supplementary Figure 1.doc]

**Supplementary Figure 1:** 10 MB region and conserved synteny of DAOA
